# Health risk realization versus warning: Impact on lifestyle behaviours

**DOI:** 10.1371/journal.pone.0338311

**Published:** 2025-12-15

**Authors:** Zoey Verdun

**Affiliations:** Department of Economics, European University Institute, San Domenico di Fiesole, FI, Italy; UF: University of Florida, UNITED STATES OF AMERICA

## Abstract

Using individual-level panel data from *Understanding Society* I estimate the response to a health risk realization on a healthy lifestyle index. To overcome the endogeneity of a diagnosis, I match on initial health risks. I find individuals improve their overall lifestyle healthiness when faced with a large negative health event such as a heart attack or diabetes diagnosis, interpreted as a precise signal about their health status, whereas they do not respond to a noisier signal through solely receiving information about certain health risk factors, such as a diagnosis of high blood pressure or angina (chest pain). The drivers of the overall effect are a decrease in the number of cigarettes smoked and an increase in not drinking alcohol; there is no significant effect found for either diet or exercise. I find some heterogeneity by sex, but only when looking at individual lifestyle behaviours. Overall, the findings suggest that the realization of a health risk leads individuals to improve their lifestyle behaviours, while only a noisier signal about their health risks leads to no such change.

## Introduction

Cardiovascular disease and diabetes are among the top ten global causes of death [1], and along with obesity are currently a source of much concern, with rising costs globally, from both direct healthcare costs and indirect productivity loss [2]. Poor lifestyle choices are a driving factor. That said, evidence exists that a healthier lifestyle can prevent such chronic diseases [3]. Studies have shown that adopting a healthier lifestyle—reducing or quitting smoking, improving diet, exercising, or reducing alcohol consumption—can improve quality of life by increasing both lifespan and quality of future years [4,5]. Finally, and quite remarkably, various medical scholars have found evidence suggesting that the progress of these diseases can be stopped or in some cases even reversed through lifestyle changes [6–8].

There is little to no causal evidence on the impact of a health risk realization on lifestyle behaviours. Governments and other institutions have strongly encouraged the adoption of healthier lifestyles, often through information campaigns. Despite these efforts, the public health and economics literature suggests that information alone is often insufficient to achieve long term lifestyle changes [9,10]. This is particularly true of diet, whereas the evidence is mixed for exercise, smoking, and alcohol consumption. Nevertheless, the medical literature has evidence of individuals who *do* make successful lifestyle changes when provided with more than just information [11–13]. Several factors are associated with such changes, including obtaining new knowledge, social support, certain personality traits, and health shocks or diagnoses; this study specifically examines the impact of the latter. Finally, there is literature on whether individuals re-optimize their lifestyle habits after important life changes [14,15]; the results are mixed.

This paper has two research questions. First, what impact does a health risk realization—the diagnosis of a heart attack or diabetes—have on lifestyle behaviours (diet, exercise, smoking, and alcohol consumption). Second, how do the responses compare between receiving only a diagnosis of a realized health risk (heart attack or diabetes) *versus* receiving only a diagnosis of risk factors (high blood pressure or chest pain); the former conceptualized as a precise signal about a person’s health status, the latter a noisier signal. This paper also explores the heterogeneity of response by sex, which is of particular interest given the differences between men and women both in the biological and social determinants and consequences of chronic disease and lifestyle behaviours [16]. In line with public health usage, *risk factors* are conditions that increase the likelihood of disease [17], while a *realized health risk*—sometimes referred to as a *health outcome* in epidemiological literature—refers to when that likelihood materializes as a diagnosis.

In the economics literature there are only a few studies investigating what lifestyle changes, if any, individuals undertake following a health related diagnosis. Furthermore, there is no consensus on which lifestyle behaviours change. Oster [18], Bhalotra et al. [19] and Hut and Oster [15] are the most related, each investigates either a response to a diagnosis of a realized health risk or a risk factor. Similarly, there are few studies in the medical literature investigating behavioural responses to a disease diagnosis [20,21]. My paper also relates to the economics literature on perceived risks and subjective probabilities of (realized) health risks [19,22]; my contribution is studying *both* the impact of the health risk being realized (clearer signal) and the impact of receiving a diagnosis for a health risk factor (noisier signal) on changes in lifestyle behaviour. This paper also contributes to the (economics) behavioural change literature, which finds that behavioural change is often difficult to achieve [9,18,19,23–26]. A more detailed literature review is provided in S1 Section 2.

This paper finds a positive association between a diagnosis of a realized health risk and a subsequent change in lifestyle; the main drivers relating to smoking and alcohol behaviours. Additionally, a heterogeneity by sex analysis finds that while the average effects are similar in magnitude and not statistically different, when the healthy lifestyle index’s effect is decomposed into its components, it turns out women respond with larger effects and across more behaviours than men. Finally, a realized health risk (heart attack or diabetes) has a large and significant effect on improving overall lifestyle behaviour, whereas the overall effect of only receiving a diagnosis of certain health risk factors is little to none.

## Data

I use data from *Understanding Society* (UKHLS), a longitudinal nationally representative survey of approximately 40,000 households in the United Kingdom. The study is managed by the Institute for Social and Economic Research (ISER) at the University of Essex and is widely regarded as a high-quality source of data for research on health, lifestyle, and socio-economic outcomes, including information on health conditions such as a diagnosis of heart attack or diabetes. While the survey covers over 80,000 individuals, only around 40,000–45,000 are consistently observed across the relevant waves used in this analysis. The final analytical sample of 15,853 individuals reflects a series of necessary exclusions, discussed in the following section on methods.

Descriptive statistics for demographics and pre-treatment lifestyle behaviours are reported in Table 1. In the sample, 44% of individuals have a bachelor’s degree or above, about half have some form of high school degree and 10% have no qualifications. The sample consists of 89% white individuals, is nearly 60% female and has an average age of 48. In terms of health behaviours, individuals, prior to treatment, on average consume 3.4 daily servings of fruits and vegetables, walk 15.4 days per month at least 10 minutes per day, walk 9.5 days per month at least 30 minutes per day, 81% do not smoke and only 12% do not drink. In the full sample, the average number of daily cigarettes smoked is 2.4, whereas among smokers the average is 11.7. For alcohol consumption, in the full sample, individuals consume 2.8 drinks on their heaviest drinking day in a week, whereas looking only at drinkers, it is 3.1. Finally, in the full sample, individuals abstain from drinking 5 out of 7 days per week, whereas drinkers abstain 4.8 days.

**Table 1:** Descriptive statistics.

	count	mean	sd
**Demographics**			
Education: GCSE or other school qualification	15,853	0.28	0.45
Education: A-level etc	15,853	0.18	0.38
Education: Bachelor’s degree or above	15,853	0.44	0.50
Non-white	15,853	0.11	0.31
Female	15,853	0.59	0.49
Age	15,853	47.84	16.07
**Health Behaviours (pre-treatment)**			
Number of servings of fruit/veg consumed per day	15,853	3.38	1.58
Number of days walked at least 10 minutes, past 4 weeks	15,853	15.43	10.80
Number of days walked at least 30 minutes, past 4 weeks	15,853	9.47	10.12
Does not smoke	15,853	0.81	0.39
Number of cigarettes smoked per day (for all)	15,853	2.38	6.20
Number of cigarettes smoked per day (for smokers)	3,234	11.69	8.94
Does not drink (at least in past 12 months)	15,853	0.12	0.32
Total drinks on heaviest drinking day, past 7 days (for all)	15,853	2.82	3.66
Total drinks on heaviest drinking day, past 7 days (for drinkers)	14,311	3.12	3.73
Number days did not drink, past 7 days (for all)	15,853	5.04	2.08
Number days did not drink, past 7 days (for drinkers)	14,311	4.83	2.08

## Methods

A pre-analysis was submitted and published on the American Economic Association’s RCT registry in October 2019 (reference: AEARCTR-0004943), for deviations see S1 Section 1.

The first five waves (2009-2015) provide pre-, during and post-treatment waves. For both the main and secondary analyses the dependent variable is a healthy lifestyle behaviour index, henceforth lifestyle index, that captures equally four lifestyle behaviours: diet, exercise, smoking and alcohol consumption. Due to limited diet and exercise related variables, fruit and vegetable consumption is used as a proxy for diet, and walking-related variables as a proxy for exercise.

The index is calculated, following Kling et al. [27], at the individual level (*i*) using an equally-weighted sum of the z-scores of the four lifestyle behaviours: diet (*B*_1_), exercise (*B*_2_), smoking (*B*_3_), and alcohol consumption (*B*_4_),


$$Index_{i}=\sum_{k=1}^{4}B_{ik}$$

where each behaviour is an average of the z-scores (*z*_*j*_) of the one to three behaviour variables (*j*) that make up that lifestyle behaviour: $B_{1}=z_{1}$, $B_{2}=(1/2)(z_{2}\;+\;z_{3})$, $B_{3}=(1/2)(z_{4}\;+\;z_{5})$, and $B_{4}=(1/3)(z_{6}\;+\;z_{7}\;+\;z_{8})$ (see Table 2). The z-score for each behaviour variable for each individual (*z*_*ij*_) is obtained by subtracting the mean of that variable ($\mu_{j}$) from the individual’s observed behaviour of that variable (*x*_*ij*_) and then dividing it by that variable’s standard deviation ($\sigma_{j}$): $z_{ij}=(x_{ij}-\mu_{j})/\sigma_{j}$.

**Table 2:** Four lifestyle behaviours consisting of eight lifestyle variables.

Diet	Walking	Smoking	Drinking Alcohol
*z*_1_: Number of servings of fruit and vegetables consumed (per day)	*z*_2_: Nr of days walked at least 10 mins (past four weeks)	*z*_4_: Dummy if currently smokes (extensive margin)	*z*_6_: Dummy if currently drinks (at least one alcoholic drink in past 12 months) (extensive margin)
	*z*_3_: Nr of days walked at least 30 mins (past four weeks)	*z*_5_: Nr of cigarettes smoked (per day) (intensive margin)	*z*_7_: Number of days did not drink alcohol (past 7 days) (intensive margin)
			*z*_8_: Number of drinks consumed on heaviest drinking day (past 7 days) (intensive margin)

All eight lifestyle behaviour variables that comprise the index are available only in waves 2, 5, and 7. However, wave 7 is excluded due to changes in key variables that make it inconsistent with waves 2 and 5. These two waves are the only ones where all four behaviours (diet, exercise, smoking and drinking) are measured consistently. While some individual behaviour variables are available in later waves, the original diet-related questions were only collected in waves 2 and 5. Therefore, extending the analysis beyond these waves would compromise the consistency of the lifestyle index. For this reason, the analysis is restricted to waves 2 and 5. Wave 1 captures any diagnoses that may have occurred prior to the start of the UKHLS. Although each wave collection period spans 24 months, at the individual-level waves are spaced 12 months apart as individuals are interviewed annually at the same time each year. Thus, the time from the measurement of the pre-diagnosis behaviour to the diagnosis is typically 0-24 months, and between the diagnosis and the post-diagnosis behaviour measurement is usually 12-36 months.

### Estimation

The analyses use first-differences (here the term “first-differences” refers to the difference between waves 2 and 5) for two reasons: (i) because the outcome variable represents a difference between two periods, and (ii) because the use of fixed effects controls for unobservable time-invariant individual heterogeneity. Since only two periods are compared, using first-differences is equivalent to using fixed effects. In each of the equations in the following two subsections, *Δ* represents the difference (or change) between the values in wave 5 and wave 2. Additionally, when analysing the individual effects of the lifestyle index’s eight variables, I apply the necessary correction to control the Type I error rate given the testing of multiple hypotheses. To this end, I use the Benjamin–Hochberg correction procedure (henceforth, Hochberg correction); it generates a critical value for each outcome to test whether that outcome is statistically significant at the 5% level. The critical values are calculated using a 0.1 false discovery rate (FDR). This method was chosen over more conservative alternatives such as Bonferroni or Holm–Bonferroni, as it controls the FDR rather than the family-wise error rate, resulting in greater statistical power. Moreover, the procedure is valid under the non-negative dependence observed among the outcomes in this analysis.

While some lifestyle behaviours, such as smoking and alcohol consumption, include both extensive and intensive margin variables, I do not apply a two-step Heckman model. This decision reflects three considerations: (i) the primary focus is on the lifestyle index, with decomposition analyses serving a secondary, interpretive role; (ii) the behavioural variables are modelled as changes between two time points, making the application of a selection model less straightforward in this context; (iii) the empirical strategy is intentionally kept consistent across behaviours to avoid introducing complexity selectively and to preserve comparability.

### Main analysis

In the main analysis, the independent variable, the diagnosis or ‘treatment’, is that of a heart attack or diabetes, where this diagnosis does not exclude the possibility of getting a diagnosis of a health risk factor (high blood pressure or chest pain) in the same time period. Only 1% of diagnosed individuals receive both diagnoses. While the effect of experiencing two diagnoses may plausibly be stronger, this study treats receiving both the same as if just receiving one. The diagnosis variable takes a value of 1 if the individual was “newly diagnosed” with at least one of either a heart attack or diabetes or 0 if not newly diagnosed with either condition. A medical condition is considered newly diagnosed if the individual responded “yes” to being diagnosed in waves 3 or 4 and “no” in the previous waves (1 and 2); A medical condition is considered as “not diagnosed” if the individual responded “no” in all four waves (1 through 4). Since these realized health risk diagnoses are relatively rare, for reasons of power, they are pooled together and treated as one. For a discussion on statistical power, small sample of ‘treated’ individuals, and minimum detectable effect size calculations, see S1 Section 3.

To evaluate the effects of the realized health risk diagnosis, I estimate the following model:


$$\Delta Index_{it}=\beta \Delta RealizedDiagnosis_{it}+\Delta u_{it}$$

where $\Delta Index_{it}$ is the change in the lifestyle behaviour index; $\Delta RealizedDiagnosis_{it}$ is the change in the independent variable, the occurrence (or not) of a diagnosis; and $\Delta u_{it}$ is the differenced error term.

Additionally, the main analysis is extended to explore heterogeneity by sex. For other heterogeneous effects, which were not specified in the pre-analysis plan (by sex, education, wave, or heart attack vs diabetes), see Section Exploring additional heterogeneous effects for results.

### Secondary analysis

The secondary analysis splits individuals into receiving exclusively *either* a diagnosis of a realized health risk *or* a diagnosis of certain health risk factors. This differentiation is important because the former gives a clear health status signal, whereas the latter is less precise; a significant share of individuals receive one or both of these risk factor diagnoses but do not later receive either a heart attack or diabetes diagnosis. To reduce confusion throughout this paper, given that both the main and secondary analyses use the diagnosis of a realized health risk but, respectively, without or with restricting a previous or concurrent diagnosis of a risk factor, when referring to the secondary analysis the term “realized health risk diagnosis” is always prefaced or followed by the word “only”.

The secondary analysis involves two regressions, estimating the effects separately for the “realized diagnosis only” and “risk factor diagnosis only” cases, each relative to receiving no diagnosis. The former equals 1 if the individual received only a diagnosis of a realized health risk and did not receive a risk factor diagnosis. The latter equals 1 if the individual received a risk factor diagnosis and did not experience a realized health risk diagnosis. For either variable, it equals 0 if neither diagnosis occurred, and individuals are excluded if they experience both kinds of diagnosis.

#### Realized diagnosis only.

To evaluate the effects of only experiencing a health risk realization, I estimate the following model:


$$\Delta Index_{it}=\beta \Delta RealizedOnly_{it}+\Delta u_{it}$$

where the variables are described as above except for the independent variable, $\Delta RealizedOnly_{it}$, which is now the difference in the ‘realized health risk diagnosis only’ variable.

#### Risk factor diagnosis only.

To evaluate the effects of only receiving a risk factor diagnosis, I estimate the following model:


$$\Delta Index_{it}=\beta \Delta RiskFactorOnly_{it}+\Delta u_{it}$$

where the variables are described as above except for the independent variable, $\Delta RiskFactorOnly_{it}$, which is now the difference in the ‘risk factor diagnosis only’ variable.

### Endogeneity, controls, and exclusion criteria

An individual’s likelihood of receiving any health diagnosis is not exogenous, it correlates with their pre-diagnosis behaviours, such as their previous diet, exercise, smoking behaviour and possibly alcohol consumption. Therefore, I account for an individual’s initial diagnosis risk level (i.e. the probability of being diagnosed with either a heart attack or diabetes) by controlling for the initial risk level using age, sex, high blood pressure, smoking (extensive and intensive margins), fruit and vegetable consumption, and physical activity. The secondary analysis excludes high blood pressure from the initial risk factors since it is part of the outcome variable. Alcohol is excluded as a risk factor in this paper. The choice is empirically grounded in guidance from public health associations, such as the American Heart Association (AHA) [28], which does not provide a consistent or definitive stance on alcohol as a risk factor for heart attack or diabetes. These initial risk factors come from risk assessment tools such as “Your Disease Risk” [29] and organizations such as the AHA [30]. Body Mass Index (BMI) and cholesterol are not included due to a high proportion of missing values (see S1 Section 1). Other controls include education, ethnicity, employment status (as a proxy for income, due to difficulties with the income data), rural/urban, and UK Government Office Regions.

The exclusion criteria are as follows: (i) Exclude individuals who have been diagnosed with a heart attack or diabetes previously (i.e. prior to wave 3). (ii) Exclude individuals if information about their diagnosis, or lack thereof, is unavailable in prior waves, to prevent confounding effects. This includes individuals for whom it is unknown whether they were diagnosed before the first wave of observation; in wave 1 individuals are asked if they were ever previously diagnosed. (iii) Exclude individuals from the control group if they live in a household with a member in the treated group (i.e. someone who has received a diagnosis), as detailed in S1 Section 1. (iv) Finally, exclude individuals if the diagnosis variable, any of the index variables, or any of the main controls are missing. For the secondary analysis an analogous set of exclusion criteria is applied, but for individuals with any previous diagnosis of high blood pressure or chest pain (angina).

Regarding attrition, given that individuals missing the diagnosis variable, any index variable, or any of the main controls are excluded, individuals missing any variables from wave 5 are also excluded. Attrition is not a concern, as only six individuals were diagnosed and then died before wave 5 data collection, and one of them was already excluded due to missing data.

### Propensity score matching

Both the main and secondary empirical analysis use kernel matching based on an estimated propensity score, comparing the average treatment effect on the treated (ATT) for treated and control units, using the first-differencing method. I estimate the propensity score using ethnicity, education, employment status, urban/rural dummy, a categorical variable for regions and the previously described initial risk factors; and bootstrap the standard errors. Table 3 reports for both demographics and pre-treatment lifestyle behaviours (including the index) no statistically significant differences between the matched treated and control groups. Of note, the alcohol consumption variables are not included in the propensity score matching (PSM) algorithm and yet are also not statistically different across groups. This observation suggests that the matching procedure works not just for observables, but also for unobservables in the sense that some non-matched variables are also “matched” without being included in the matching procedure. The propensity score estimations satisfy the three necessary conditions: balancing property, unconfoundedness assumption and common support.

**Table 3:** Summary statistics—Comparing matched treated and controls.

Variable	Mean		p (t-test)
	Treated	Control	
**Demographics**			
Age	59.41	58.08	0.350
Female	0.46	0.49	0.543
Non-white	0.09	0.12	0.185
Education: No qualification	0.22	0.22	0.935
Education: GCSE or other school qualification	0.34	0.33	0.956
Education: A-level etc	0.15	0.16	0.797
Education: Bachelor’s degree or above	0.29	0.29	0.941
Urban	0.24	0.25	0.822
Employment status: Inactive	0.66	0.65	0.785
Employment status: PT Employed	0.11	0.11	0.913
Employment status: FT Employed	0.23	0.24	0.822
**Health Behaviours (pre-treatment)**			
Number of servings of fruit/veg per day	3.20	3.24	0.780
Number of days walked at least 10 min, past 4 weeks	12.87	13.65	0.451
Number of days walked at least 30 min, past 4 weeks	7.83	8.03	0.825
Does not smoke	0.76	0.76	0.925
Number of cigarettes per day	3.52	3.28	0.738
Does not drink (past 12 months)	0.13	0.14	0.653
Total drinks on heaviest drinking day	2.39	2.58	0.562
Number days did not drink, past 7 days	5.25	4.93	0.132
**Pre-treatment Healthy Lifestyle Index Score**			
Healthy Behaviour Index	–0.45	–0.41	0.835

Note: None of the differences between treated and control are statistically significant at the 5% level. For the smoking and alcohol variables the full sample is used, regardless of whether individuals smoke or drink.

### Ethics statement

The data were accessed for research purposes on 26 December 2018. The author did not have access to information that could identify individual participants during or after data collection. Ethical approval was not required.

## Results

### Main analysis

**Index.** The effect of a realized health risk diagnosis on changes to the lifestyle index is reported in Table 4 (Column 1), showing an increase of 0.227 units in the index. This suggests that an individual who receives a realized health risk diagnosis improves their lifestyle, with one or more lifestyle behaviours becoming healthier. Although not part of the pre-analysis plan, a significant difference is found at the 10% level between older and younger treated individuals (see Section Exploring additional heterogeneous effects). To better understand which and how many of the index’s lifestyle behaviours are driving the result, the effects of the individual behaviour variables are reported in columns 2-9 of Table 4.

**Table 4:** Realized health risk diagnosis: ATT of change in lifestyle index and components.

	Index	Fruit/Veg	Walk 10	Walk 30	Smoke	Nr Cigs	Drink	Heavy	Days
Realized Diagnosis	0.227^**^	0.222^*^	–1.489^*^	–1.326^*^	0.0361^*^	4.525^*** + ^	0.0632^** + ^	0.174	0.0836
	(0.111)	(0.122)	(0.853)	(0.736)	(0.0193)	(1.582)	(0.0254)	(0.223)	(0.129)
Observations	15,853	15,853	15,853	15,853	15,853	3,234	15,853	14,311	14,311

Realized health risk diagnosis: heart attack or diabetes diagnosis in wave 3 or 4. Independent variable: difference between the realized health risk diagnosis variable in wave 2 (pre-treatment) and wave 5 (post-treatment). Each dependent variable: difference between its value in wave 2 (pre-treatment) and wave 5 (post-treatment). Bootstrap standard errors in parentheses, 1000 reps. Kernel matching using 0.0075 bandwidth for index and 0.0009375 bandwidth for individual index components. ^*^ *p* < 0.1, ^**^ *p* < 0.05, ^***^ *p* < 0.01, ^ +^ *p* < 0.05 using a Hochberg correction with FDR of 0.1 (correction not applicable for index coefficient).

To aid interpretation of the effect size, a 0.227-unit increase in the lifestyle index corresponds, for example, to an increase of approximately 0.36 daily servings of fruits and vegetables, assuming all other lifestyle behaviours remain unchanged. For context, the UK’s “5-a-day” information campaign led to an average increase of 0.3 servings of fruits and vegetables per day [31], and a review of interventions in Western countries found an average increase of 0.6 servings [32]. These figures suggest that the magnitude of the observed effect is in line with previous findings in the literature.

**Individual behaviour variables.** Within the remaining eight columns of Table 4 a positive coefficient is interpreted as: i) an increase in the quantity of a healthy behaviour, ii) a decrease in the quantity of an unhealthy behaviour, or iii) a quitting of an unhealthy behaviour between waves 2 and 5.

The key drivers behind the effect of the index are a decrease in the daily number of cigarettes smoked (4.53 cigarettes, 1% significance level) and an increase in not drinking alcohol (6.32 percentage points, 5% significance level). Possible, but less precisely estimated, drivers include an increase in fruit and vegetable consumption (0.22 servings), an increase in smoking cessation (3.61 percentage points), and decreases in the number of days per month individuals walked at least 10 minutes (1.49 days) and at least 30 minutes (1.33 days). The negative point estimates for the days walked variables hint at possible physical limitations from experiencing a negative health event, such as suffering from a heart attack or diabetes.

Both the decrease in the number of cigarettes smoked and the increase in the likelihood of not drinking alcohol remain statistically significant after applying the multiple hypothesis correction. However, while fruit and vegetable consumption, smoking cessation, and the days walked variables are statistically significant at the 10% level, they no longer remain significant after the correction. As for alcohol consumption, the reduction in drinks consumed on the heaviest drinking day and the number of days an individual abstained from drinking are not statistically significant, but suggest a possible decrease in alcohol consumption frequency and intensity.

### Main analysis—Heterogeneity by sex

**Index.** The heterogeneous effect of a realized health risk diagnosis on the index by females and males is reported in Column 1 of Tables 5 and 6, respectively. The effect for women is a 0.22 unit increase in the index, slightly lower than the statistically significant 0.25 unit increase for men; however, the difference is not statistically significant.

**Table 5:** ATT of change in lifestyle index and components, by sex—Female.

	Index Female	Fruit/Veg Female	Walk 10 Female	Walk 30 Female	Smoke Female	Nr Cigs Female	Drink Female	Heavy Female	Days Female
Realized Diagnosis	0.220	0.220	–2.493^** + ^	–2.288^** + ^	0.0771^** + ^	3.857	0.138^*** + ^	0.130	0.00279
	(0.180)	(0.163)	(1.211)	(1.109)	(0.0313)	(2.470)	(0.0434)	(0.266)	(0.171)
Observations	9,311	9,311	9,311	9,311	9,311	1,857	9,311	8,266	8,266

Realized health risk diagnosis: heart attack or diabetes diagnosis in wave 3 or 4. Independent variable: difference between the realized health risk diagnosis variable in wave 2 (pre-treatment) and wave 5 (post-treatment). Each dependent variable: difference between its value in wave 2 (pre-treatment) and wave 5 (post-treatment). Bootstrap standard errors in parentheses, 1000 reps. Kernel matching using 0.00046875 bandwidth for index and 0.0009375 bandwidth for individual index components. ^*^ *p* < 0.1, ^**^ *p* < 0.05, ^***^ *p* < 0.01, ^ +^ *p* < 0.05 using a Hochberg correction with FDR of 0.1 (correction not applicable for index coefficient).

**Table 6:** ATT of change in lifestyle index and components, by sex—Male.

	Index Male	Fruit/Veg Male	Walk 10 Male	Walk 30 Male	Smoke Male	Nr Cigs Male	Drink Male	Heavy Male	Days Male
Realized diagnosis	0.248^*^	0.287^*^	–0.605	–0.361	0.00347	5.190^**^	0.00301	0.242	0.155
	(0.138)	(0.170)	(1.172)	(1.008)	(0.0216)	(2.158)	(0.0281)	(0.344)	(0.181)
Observations	6,542	6,542	6,542	6,542	6,542	1,377	6,542	6,045	6,045

Realized health risk diagnosis: heart attack or diabetes diagnosis in wave 3 or 4. Independent variable: difference between the realized health risk diagnosis variable in wave 2 (pre-treatment) and wave 5 (post-treatment). Each dependent variable: difference between its value in wave 2 (pre-treatment) and wave 5 (post-treatment). Bootstrap standard errors in parentheses, 1000 reps. Kernel matching using 0.005625 for index and 0.0009375 bandwidth for individual index components. ^*^ *p* < 0.1, ^**^ *p* < 0.05, ^***^ *p* < 0.01, ^ +^ *p* < 0.05 using a Hochberg correction with FDR of 0.1 (correction not applicable for index coefficient).

**Individual behaviour variables.** A decomposition of the effect into the index’s eight lifestyle behaviour variables provides insights into the behavioural differences between women and men, reported in Columns 2-9 of Tables 5 and 6, respectively. The only statistically significant differences between women and men are in smoking cessation (10% significance level) and quitting alcohol consumption (1% significance level).

However, when looking at each sex separately, for women, four of the eight lifestyle behaviours exhibit statistically significant changes: a decrease in the number of days walked at least 10 and at least 30 minutes in a month, by 2.49 days and 2.29 days, respectively (both at the 5% level), alongside increases in smoking cessation by 7.71 percentage points (5% level) and quitting drinking alcohol by 13.8 percentage points (1% level). For men, none of the variables remain statistically significant after multiple hypothesis correction. However, some weaker evidence suggests potential increases in fruit and vegetable consumption by 0.29 servings (10% level) and a decrease in the number of cigarettes smoked by 5.19 cigarettes (5% level). Among smokers, the reduction in the number of cigarettes smoked corresponds to a 40.8% decrease for men and 35.3% decrease for women, relative to pre-diagnosis levels, indicating that men reduce their smoking intensity more both in absolute and relative terms. Neither sex exhibits a statistically significant decrease in alcohol drinking intensity or frequency.

Thus, while at first glance it appears that women and men react similarly to the realized health risk diagnosis, the decomposition reveals a more nuanced picture. Women react more strongly than men, but their overall behavioural response is partially offset: while they improve certain health behaviours (e.g., smoking cessation and quitting alcohol), they also experience a significant reduction in walking, which counterbalances these improvements in the index.

Finally, although not included in my pre-analysis plan, I conduct a heterogeneity analysis examining responses to a realized health risk diagnosis by type (heart attack vs. diabetes) and by sex. However, due to limited statistical power, I am unable to determine whether the difference in response between women and men for heart attacks is statistically distinct from that for diabetes.

### Secondary analysis—Realized diagnosis only

**Index.** Table 7 Column 1 reports a positive impact of a realized health risk diagnosis only on the lifestyle index: an increase of 0.534 units, statistically significant at the 1% level; suggesting that individuals respond to this diagnosis by significantly improving their lifestyle behaviours. Although not part of the pre-analysis plan, a statistically significant difference is found at the 1% level between individuals with and without some higher education (see Section Exploring additional heterogeneous effects).

**Table 7:** Realized health risk diagnosis only: ATT of change in lifestyle index and components.

	Index	Fruit/Veg	Walk 10	Walk 30	Smoke	Nr Cigs	Drink	Heavy	Days
Realized Diagnosis Only	0.534^***^	0.418^** + ^	–0.504	–1.090	0.0946^** + ^	6.340^** + ^	0.0538	0.143	0.139
	(0.194)	(0.190)	(1.246)	(1.034)	(0.0383)	(2.525)	(0.0341)	(0.261)	(0.183)
Observations	12,339	12,339	12,339	12,339	12,339	2,612	12,339	11,176	11,176

Realized health risk diagnosis only: diagnosis of heart attack or diabetes in wave 3 or 4, without a concurrent or existing diagnosis of high blood pressure or chest pain. Independent variable: difference between the realized health risk diagnosis variable in wave 2 (pre-treatment) and wave 5 (post-treatment). Each dependent variable: difference between its value in wave 2 (pre-treatment) and wave 5 (post-treatment). Bootstrap standard errors in parentheses, 1000 reps. Kernel matching using 0.00375 bandwidth for index and 0.00140625 bandwidth for individual index components. ^*^ *p* < 0.1, ^**^ *p* < 0.05, ^***^ *p* < 0.01, ^ +^ *p* < 0.05 using a Hochberg correction with FDR of 0.1 (correction not applicable for index coefficient).

**Individual behaviour variables.** The impact of receiving only the realized health risk diagnosis on the eight index variables is reported in Table 7 Columns 2-9, with and without Hochberg correction. Three variables have statistically significant effects at the 5% level and remain significant after correction: increase in fruit and vegetable consumption (0.42 servings), increase in smoking cessation (9.46 percentage points), and decrease in the number of cigarettes smoked (6.34 cigarettes). Beyond statistical significance, except for the walking variables, all point estimates have the expected non-negative sign. Given that there are fewer treated individuals in this realized health risk diagnosis only case, it is possible that decomposed there is not enough power to statistically detect all the separate effects of the index’s individual behaviour variables (see S1 Section 3 for a table of minimum detectable effect sizes).

### Secondary analysis—Risk factor diagnosis only

**Index.** The impact of receiving only a risk factor diagnosis on the lifestyle index is reported in Table 8 Column 1. In this case, there is no statistically significant increase in the index. Even when going beyond statistical significance and considering the size of the point estimate, using the 95% confidence interval, an effect size larger than 0.255 can be ruled out, which is still twice as small an effect compared to the realized health risk diagnosis only case.

**Table 8:** Risk factor diagnosis only: ATT of change in lifestyle index and components.

	Index	Fruit/Veg	Walk 10	Walk 30	Smoke	Nr Cigs	Drink	Heavy	Days
Risk Factor Diagnosis Only	0.0856	0.0230	–0.419	–0.864	0.0272^**^	1.553^*^	0.0326^**^	0.115	0.240^** + ^
	(0.0865)	(0.0833)	(0.571)	(0.529)	(0.0138)	(0.855)	(0.0160)	(0.163)	(0.0948)
Observations	12,701	12,701	12,701	12,701	12,701	2,697	12,701	11,496	11,496

Risk factor diagnosis only: diagnosis of high blood pressure or chest pain only. Independent variable: difference between risk factor diagnosis variable in wave 2 (pre-treatment) and wave 5 (post-treatment). Each dependent variable: difference between its wave 2 (pre-treatment) and wave 5 (post-treatment) values. Bootstrap standard errors in parentheses, 1000 reps. Kernel matching (0.00140625 bandwidth). ^*^ *p* < 0.1, ^**^ *p* < 0.05, ^***^ *p* < 0.01, ^ +^ *p* < 0.05 using a Hochberg correction with FDR of 0.1 (correction not applicable for index coefficient).

**Individual behaviour variables.** The impact of only a risk factor diagnosis on the eight index variables is reported in Table 8 Columns 2-9, both with and without Hochberg correction. All the point estimates have the expected positive sign except for the walking variables. Other than an increase of 0.24 days in the number of days per week individuals abstain from drinking alcohol, none of the variables have a statistically significant effect after applying the Hochberg correction. This confirms the finding that there is little to no effect of the noisier health status signal on lifestyle change.

## Discussion

The main analysis shows that individuals tend to improve their overall lifestyle following a realised health risk diagnosis, as measured by the lifestyle index composed of diet, physical activity, smoking, and alcohol consumption behaviours. While the overall effect is positive, it masks variation across behaviours. A notable example is physical activity, where a consistent decline in walking is observed, even as improvements occur in other behaviours such as drinking or smoking. As a robustness check, the analysis is also run separately by type of diagnosis and by wave of diagnosis, with no significant differences in estimated effects found across these specifications (see Section Exploring additional heterogeneous effects).

When examining responses by sex, average changes in the index are similar for men and women. However, disaggregated results show that women adjust more behaviours overall, with statistically significant differences in smoking cessation and quitting alcohol compared to men. A larger and statistically significant decline in time spent walking is observed among women. One possible explanation is that heart attacks are harder to detect in women and therefore less frequently diagnosed compared to men [33]. As a result, the heart attacks that are detected in women may be, on average, more severe, eliciting a stronger behavioural response. This may account for the observed larger reduction in physical activity, potentially reflecting increased physical limitations following more serious events.

The first part of the secondary analysis, which focuses on individuals who received only a realised health risk diagnosis, heart attack or diabetes, reveals a significant improvement in overall lifestyle behaviours. The impact is most notable in diet and smoking-related behaviours. Specifically, individuals increase their consumption of fruits and vegetables and demonstrate a significant reduction in smoking, both in terms of cessation and the number of cigarettes smoked. These findings are consistent with existing literature. For instance, Oster [18] reports a small but significant improvement in diet following a diabetes diagnosis, including increased fruit and vegetable consumption. The observed smoking cessation effect is similarly in line with Chong et al. [20], who find a significant increase in quitting after a diabetes diagnosis. While I also find a significant reduction in the number of cigarettes smoked, Chong et al. [20] do not find a statistically significant effect, though their coefficient suggests a reduction.

For the second part of the secondary analysis, which focuses on individuals with only a risk factor diagnosis, high blood pressure or chest pain, a few variables related to reduction in smoking and drinking initially showed significant findings. However, after correcting for multiple hypothesis testing, only the increase in the number of days individuals abstained from drinking alcohol remains significant. These findings are consistent with recent evidence by Bhalotra et al. [19]. While Bhalotra et al. [19] find no impact on alcohol consumption following a high blood pressure diagnosis, my results suggest a small but significant increase in days without alcohol consumption. While I find a possible increase in smoking cessation, which does not remain significant after correcting for multiple hypotheses, Bhalotra et al. [19] report a statistically significant, and larger, effect. Finally, consistent with Bhalotra et al. [19], my analysis finds no significant impact on exercise following a risk factor diagnosis.

The contrast between the two secondary analysis cases is striking. Individuals who receive only a realised health risk diagnosis (heart attack or diabetes) show a statistically significant improvement in the lifestyle index, despite the relatively small number of treated individuals, whereas those diagnosed only with a risk factor (high blood pressure or chest pain), despite a larger treated group, show no significant effect. The point estimate for the realised health risk diagnosis group is approximately six times larger and this difference is statistically significant. This substantial divergence is further supported by the decomposition of the index: for nearly every behaviour, the estimated effect of a realised health risk diagnosis is either significantly greater (i.e., healthier) or not statistically different from that of a risk factor diagnosis. While it may seem intuitive that a clearer signal of health deterioration prompts stronger behavioural responses, this paper is, to the best of my knowledge, the first to provide empirical evidence of this distinction. One possible explanation is that more precise information about one’s health leads to more accurate belief updating, thereby increasing motivation to adopt healthier behaviours.

A few limitations should be noted. First, the health behaviour variables are only observed in waves 2 and 5, restricting the ability to assess behavioural changes in between those two waves. Second, due to the relatively small number of ‘treated’ individuals for each disease, heart attacks and diabetes had to be pooled into a single realised health risk diagnosis group, limiting the ability to distinguish disease-specific effects. Third, and finally, not all relevant risk factors could be included as controls—such as BMI or cholesterol levels—due to high rates of missing data for these variables.

## Conclusions

This paper finds that individuals improve their overall lifestyle after experiencing a strong health shock by improving some of their lifestyle behaviours, as measured using a healthy lifestyle index, and that the main drivers are quitting smoking and drinking. It also empirically confirms the intuition that some individuals make lifestyle changes when the diagnosis received is more severe (heart attack or diabetes), interpreted as receiving a more precise signal about their health status, but not when the signal is less strong.

Realized health risk diagnosis: diagnosis of heart attack or diabetes in wave 3 or 4. Bootstrap standard errors in parentheses, 1000 reps. Kernel matching (0.0075 bandwidth). ^*^ *p* < 0.1, ^**^ *p* < 0.05, ^***^ *p* < 0.01.

Furthermore, although the overall effect on lifestyle changes between men and women is of similar magnitude, the heterogeneity lies in the number and size of changes made to the behaviours that comprise the lifestyle index. Women make more and larger changes, however some of these have opposite effects on the index. One reason for this sex differential may be attributable to the greater difficulty of diagnosing heart attacks in women. I also find heterogeneous effects by age and education.

This paper finds that while individuals do respond to a significant health shock, there is no such behavioural change at an earlier ‘warning’ stage (i.e. when diagnosed with high blood pressure or chest pain). This finding suggests room for policy to help encourage behavioural change earlier on when such changes are more preventative in nature rather than responsive; earlier changes could reduce or prevent the high medical costs and loss of (quality) life often accompanying such health diagnoses. Additionally, given individuals seem more inclined to modify their smoking and alcohol behaviours in response to a health shock, there is more need for policy to support changes in diet and exercise, which seem more resistant to change.

Finally, the changes to the lifestyle behaviours found in this paper are for the time horizon of one to two years after a diagnosis; it would be very interesting and societally relevant to investigate in future research if such changes are also sustained in the longer run.

## Appendix

## Exploring additional heterogeneous effects

I did not preregister any of the following heterogeneous effects in my pre-analysis plan. However, I investigate these different heterogeneous effects at the suggestion of a few readers of an earlier version of this paper.

**Split by median age of treated.** Table 9 shows the heterogeneous effects of age on the change in lifestyle index in response to a realized health risk diagnosis. Tables 10 and 11 also show the heterogeneous effects of age but now in response to either exclusively the realized health risk diagnosis or the risk factor diagnosis, respectively. What is interesting to note is that for the main analysis realized health risk diagnosis (Table 9) only the younger treated individuals respond significantly to the diagnosis; there is no significant response from older individuals; and the difference is statistically significant at the 10% level. By contrast, in the case of receiving only the realized health risk diagnosis (Table 10) it appears that both younger and older treated individuals respond equally strong. Although not an exact comparison, this finding is in line with Oster [18] who finds no effect of age on the impact of a diabetes diagnosis on diet. Finally, in the case of receiving only the risk factor diagnosis (Table 11) there is no significant reaction to the diagnosis nor a significant difference in the response between younger and older treated individuals; this is not surprising since there is no (large) response to the risk factor diagnosis in the main analysis either.

**Table 9:** Realized health risk diagnosis on index—Split by median age.

	Younger (<62 years)	Older (≥ 62 years)
Realized Diagnosis	0.452^***^	0.0391
	(0.170)	(0.145)
Observations	12,231	3,622

Realized health risk diagnosis definition: diagnosis of heart attack or diabetes in wave 3 or 4. Bootstrap standard errors in parentheses, 1000 reps. Kernel matching (0.0075 bandwidth). ^*^ *p* < 0.1, ^**^ *p* < 0.05, ^***^ *p* < 0.01.

**Table 10:** Realized health risk diagnosis only on index—Split by median age.

	Younger (≤58 years)	Older (>58 years)
Realized Diagnosis Only	0.540^**^	0.551^*^
	(0.255)	(0.313)
Observations	9,820	2,519

Realized health risk diagnosis only: diagnosis of heart attack or diabetes in wave 3 or 4, without concurrent or existing diagnosis of high blood pressure or chest pain. Bootstrap standard errors in parentheses, 1000 reps. Kernel matching (0.00375 bandwidth). ^*^ *p* < 0.1, ^**^ *p* < 0.05, ^***^ *p* < 0.01.

**Table 11:** Risk factor diagnosis only on index—Split by median age.

	Younger (≤55 years)	Older (>55 years)
Risk Factor Diagnosis Only	0.173	0.0186
	(0.131)	(0.108)
Observations	9,392	3,309

Risk factor diagnosis only definition: diagnosis of high blood pressure or chest pain. Bootstrap standard errors in parentheses, 1000 reps. Kernel matching (0.0075 bandwidth). ^*^ *p* < 0.1, ^**^ *p* < 0.05, ^***^ *p* < 0.01.

**Split by (no) higher education.** Table 12 shows the heterogeneous effects of (not) having some higher education on the change in lifestyle index in response to a realized health risk diagnosis. Tables 13 and 14 also show the heterogeneous effects of education but now in response to the realized health risk diagnosis only and the risk factor diagnosis only cases, respectively. For the case of education, the findings are clear that it is the individuals with no higher education (lower than a bachelor’s degree) that react to the realized health risk diagnosis, both in the main analysis realized health risk diagnosis case (Table 12) and the realized health risk diagnosis only case (Table 13). There are no (heterogeneous) effects for the risk factor diagnosis only case (Table 14).

**Table 12:** Realized health risk diagnosis on index—Split by (no) higher education.

	No Higher Education	Some Higher Education
Realized Diagnosis	0.294^**^	0.0603
	(0.139)	(0.177)
Observations	8,904	6,949

Realized health risk diagnosis definition: diagnosis of heart attack or diabetes in wave 3 or 4. Bootstrap standard errors in parentheses, 1000 reps. Kernel matching (0.0075 bandwidth). ^*^ *p* < 0.1, ^**^ *p* < 0.05, ^***^ *p* < 0.01.

**Table 13:** Realized health risk diag. Only on index—Split by (no) higher education.

	No Higher Education	Some Higher Education
Realized Diagnosis Only	0.717^***^	0.114
	(0.262)	(0.254)
Observations	6,540	5,799

Realized health risk diagnosis only: diagnosis of heart attack or diabetes in wave 3 or 4, without concurrent or existing diagnosis of high blood pressure or chest pain. Bootstrap standard errors in parentheses, 1000 reps. Kernel matching (0.00375 bandwidth). ^*^ *p* < 0.1, ^**^ *p* < 0.05, ^***^ *p* < 0.01.

**Table 14:** Risk factor diagnosis only on index—Split by (no) higher education.

	No Higher Education	Some Higher Education
Risk Factor Diagnosis Only	0.103	0.0530
	(0.109)	(0.150)
Observations	6,767	5,934

Risk factor diagnosis only: diagnosis of high blood pressure or chest pain. Bootstrap standard errors in parentheses, 1000 reps. Kernel matching (0.0075 bandwidth). ^*^ *p* < 0.1, ^**^ *p* < 0.05, ^***^ *p* < 0.01.

Comparing my findings to that of the literature, my realized health risk diagnosis only finding is not in line with Oster [18], who finds no effect of demographics (including education) on the impact of diet on a diabetes diagnosis. However, in my analysis the effect of the realized health risk diagnosis only is being driven not just by diet, but also by smoking behaviour (both the number of cigarettes and smoking cessation), therefore it is not an ideal comparison. In the case of the risk factor diagnosis only, my finding is in line with Bhalotra et al. [19], who study the impact of high blood pressure on different lifestyle behaviours, they also do not find heterogeneous effects by education.

**Split by timing of diagnosis.** Recall that the timeline of the data is pre-diagnosis lifestyles are measured in wave 2, realized health risk diagnoses (or risk factor diagnoses) occur in wave 3 and/or 4, and post-diagnosis lifestyles are measured in wave 5. The longer/shorter time since the diagnosis relative to the post-diagnosis moment of measurement of lifestyle change has two mechanisms that can affect the strength of the response in opposing manners. On the one hand an earlier occurring diagnosis (wave 3) may give individuals more time to adopt lifestyle changes, in which case I expect a larger effect for individuals who experienced a diagnosis in wave 3 compared to wave 4. However, on the other hand, it could also be that the initial changes made after the diagnosis are not sustained in the longer run and therefore the (longer term) effect of a diagnosis in wave 3 on lifestyle change is weaker than a diagnosis that occurred in wave 4. I omit individuals who get a diagnosis in both waves, which is less than 10% of the treated sample. Table 15 shows the heterogeneous effects of experiencing a diagnosis in wave 3 versus wave 4. These findings suggest the first mechanism plays a larger role—individuals need sufficient time to change their lifestyle after a diagnosis rather than that they lose (some of) their initial changes made over time.

**Table 15:** Realized health risk diagnosis on index—Split by timing of diagnosis.

	Diagnosed in Wave 3	Diagnosed in Wave 4
Realized Diagnosis	0.309^*^	0.187
	(0.187)	(0.155)
Total Observations	15,713	15,738
Treated Observations	92	117

Realized health risk diagnosis: diagnosis of heart attack or diabetes in wave 3 or 4. Bootstrap standard errors in parentheses, 1000 reps. Kernel matching (0.0075 bandwidth). ^*^ *p* < 0.1, ^**^ *p* < 0.05, ^***^ *p* < 0.01.

**Split by realized health risk diagnosis.** Recall that the main realized health risk diagnosis variable consists of individuals who have had either a heart attack and/or a diabetes diagnosis, and that the main reason for pooling these diagnoses is to increase statistical power. Although both diagnoses require a similar change in lifestyle to alleviate or even reverse the condition, individuals may respond differently across the diagnoses. Table 16 investigates any heterogeneous effects across the two diagnoses. I omit individuals who experience both diagnoses, about 1% of the treated sample.

**Table 16:** Realized health risk diagnosis on index—Diagnosis split by medical condition.

	Heart Attack	Diabetes
Realized Diagnosis	0.270	0.180
	(0.193)	(0.136)
Total Observations	15,695	15,776
Treated Observations	74	155

Realized health risk diagnosis: diagnosis of a realized health risk in wave 3 or 4. Each column represents a diagnosis of that realized health risk only. Bootstrap standard errors in parentheses, 1000 reps. Kernel matching (0.0075 bandwidth). ^*^ *p* < 0.1, ^**^ *p* < 0.05, ^***^ *p* < 0.01.

## Supporting information

S1 Web Appendix Lorem ipsum. (PDF)
