# Supplementary material for: Health risk realization versus warning: Impact on lifestyle behaviours
Source: PLoS One. 2025 Dec 15;20(12):e0338311. doi: 10.1371/journal.pone.0338311 (PMC12704847; doi:10.1371/journal.pone.0338311)
Supplement: S1 Web Appendix — (PDF) [file pone.0338311.s001.pdf]

S1 Web Appendix

1 Deviations from Pre-Analysis Plan

The location of the plan is: https://www.socialscienceregistry.org/trials/4943/history/56027. The following sections discuss the deviations.

1.1 Additional Outcome Variable

My pre-analysis plan mentions seven health behaviour outcome variables, whereas this paper uses eight. The additional outcome variable captures the extensive margin of alcohol consumption: a dummy for if an individual is a drinker or not. Furthermore, the original pre-analysis plan did not include that I would summarize these behaviours into a lifestyle index. I introduced an index to have only a single outcome variable.

The reason for including this additional alcohol consumption variable is the lack of an extensive margin variable for alcohol consumption. In the case of the smoking variables there is one intensive and one extensive margin variable. In the case of alcohol consumption, there were only two intensive margin variables. However, I realized it is important to have both an extensive and an intensive margin variable for this behaviour as it is possible that individuals behave heterogeneously after experiencing a diagnosis (whether it be for a realized health risk or a risk factor), changing one of these margins but maybe not both.

Finally, I keep both the intensive margin variables for alcohol consumption: the number of days an individual did not drink alcohol in the past seven days and the total number of drinks an individual consumed on the heaviest drinking day in the past seven days. The reason is that the literature on harmful alcohol consumption and behaviours suggests that when measuring the most harmful aspects of alcohol consumption on health (i.e. binge drinking) it is *both* the intensity with which an individual drinks in a given period of time (about two hours) and how often per week an individual drinks that matter.

1.2 No Splitting of Pooled Realized Health Risk or Risk Factor Variables

The separating of the pooled realized health risk diagnosis (or risk factor diagnosis) variable, as a robustness check, for either the main analysis or secondary analysis is not performed, though I mentioned it in the pre-analysis plan. The reason is insufficient power, the main reason to pool to begin with. However, I do run the first regression of the main analysis split by diagnosis as a robustness check to confirm the effect magnitudes are similar, see Table 16.

1.3 No BMI or Blood Cholesterol in Propensity Score

In the pre-analysis plan I wrote that if more than half the sample had the BMI and blood cholesterol variables non-missing, I would perform a robustness check to see if there is any impact of their inclusion on the final findings. However, for the blood cholesterol variable only 32% of the sample is

non-missing and for BMI only 48% is non-missing. Therefore, these variables are not included in any analysis or robustness check.

1.4 Changes to Empirical Strategy

The pre-analysis plan specified I would match the treated with the controls in my sample and then do regression analysis on that matched sample, either matching using stratification or nearest neighbour 1:1. However, given that kernel matching provides the best balance, I use it instead. Therefore, instead of matching treated with control units and then running standard regression analyses using the matched sample, I use the matching commands directly. This means that the matching program will match the treated and controls using the selected strategy and then provide the ATT (or ATE, if desired). The consequence of changing the technical approach to regression analysis it is no longer possible to include household clustered standard errors (something I said I would do in my pre-analysis plan). In theory, the inclusion of household clustered standard errors is considered important because there are individuals in the sample that live in the same household together. It is important to take into account that such individuals may not be identically and independently distributed. One example of this possible dependence is if one household member receives a diagnosis another household member may adjust their behaviour, even without having received a diagnosis themselves. I can include the clustered standard errors in a regular regression analysis, but cannot when using matching analysis commands directly. However, the use of a different matching strategy and/or different standard errors have no effect on the findings, as discussed in Matching and Weighting Strategies.

Furthermore, to address the likely lack of independence between household members, I omit all household members who would be classified as a control individual but who are part of a household that contains a treated individual. This is because other individuals inside the same household as a treated individual, especially in the case of heart attack, often improve their lifestyle behaviours significantly and persistently (Fadlon and Nielsen, 2019). Such omitted "treated-control" members make up less than 1% of the sample.

1.5 Multiple Hypothesis Correction

My pre-analysis plan stated the use of the Holm-Bonferroni procedure. The reasoning was that the more powerful Hochberg procedure only holds under non-negative dependence. However, that reasoning was incorrect since the correlations between the outcomes are all non-negative. Most of the correlations between the outcomes are positive and the select few that have a negative sign have such a small correlation coefficient that I consider those to be essentially zero. Therefore, ex-post I decided the use of the Benjamin-Hochberg correction procedure, henceforth Hochberg correction, instead was better; it is uniformly more powerful than the Holm-Bonferroni procedure.

2 Related Literature

The majority of the health economics literature on behavioural change builds upon the notion that the provision of (new) information, whether it be generic or tailored to the individual, and the subsequent updating of beliefs influences behaviour. However, a growing body of literature, not just in economics but also in health and psychology, finds that knowledge or information is usually necessary but not sufficient for change (Bartiaux, 2008; Mathis and Steffen, 2015; Kelly and Barker, 2016). For example, Kelly and Barker (2016) highlight several common errors made when attempting to foster behavioural change that relate to information, whether it be changes in diet, alcohol consumption or physical activity. Two such errors are assuming "knowledge and information drive behaviour" and "it is about getting the message across" (p.111). There is also a large body of literature in behavioural economics that looks at behavioural biases and self-control issues such as time-inconsistent and present-biased preferences (see for example Laibson, 1997; O'Donoghue and Rabin, 1999, 2015; Benhabib et al., 2010).

That being said, when looking across different individual health behaviours the evidence of the impact of information on behavioural change is mixed. For diet, it is the most clear, generic information is not sufficient (Brownell and Cohen, 1995; Worsley, 2002). There is even evidence that individuals are willing to forgo health benefits to prevent having to modify their diet (Atkin, 2016; Oster, 2018). For exercise, Young et al. (1996) find a health education campaign has little to no effect on physical activity levels. In contrast, Craig et al. (2006) find a public-awareness campaign to increase walking among adults, using an objective self-monitoring tool, to be effective. For smoking, two integrative reviews find mass media campaigns effective when it comes to fostering awareness and behavioural change (Flay, 1987; Durkin et al., 2012). However, Strecher et al. (1994), using computer-tailored targetted messaging, find positive effects only for light to moderate smokers. Finally, for alcohol consumption Fleming et al. (1999) find interventions by a physician can be effective in reducing different outcomes of alcohol use. In contrast, a review of the existing literature on prevention and treatment of college students finds little support for educational or awareness programs, but more support for other (non-informational) interventions in reducing alcohol use and its negative consequences (Larimer and Cronce, 2002).

Compared to the limited success of broader information campaigns there are more examples of successful change in both the behavioural change literature and the medical literature. However, these examples often involve more than just the general provision of information. Significant behavioural change, when it happens, often occurs in specific subgroups of individuals or individuals in certain situations. For example, in the economics literature, Hut and Oster (2022) find evidence that dietary change only occurs in a specific sub-group of individuals, those whose baseline diet consists of a small number of foods. In the medical literature, such specific groups of individuals or situations are usually patients of certain doctors, intensive programs or interventions (Esselstyn et al., 1995; Lanza et al., 2001). For example, in the Lifestyle Heart Trial patients sustained intensive lifestyle changes — diet, exercise, smoking and stress management — over several years (Ornish et al., 1998).

There is also a growing literature in economics on the impact of the diagnosis of risk factors, such as hypertension or high blood pressure, and the provision of (tailored) health status information on

changes in dietary behaviour (Zhao et al., 2013; Belot et al., 2020; Carrera et al., 2020) and other lifestyle behaviours such as smoking, exercise and alcohol use (Edwards, 2018; Bhalotra et al., 2020). In this literature, the paper closest to mine is Bhalotra et al. (2020). They find a high blood pressure diagnosis leads to a reduction in smoking but it has no impact on either exercise or alcohol consumption; furthermore, they find possible but imprecise evidence that diets marginally improve after diagnosis. My paper differs from Bhalotra et al. (2020) in that where they only look at the impact of a risk factor diagnosis, high blood pressure, I look at the diagnosis of risk factors *and* the corresponding diagnosis of the realized health risk itself.

Regarding the other related papers, Zhao et al. (2013) studies the impact of a high blood pressure diagnosis on diet and finds that individuals reduce fat intake, with richer individuals reducing more. Carrera et al. (2020) look at changes in workplace cafeteria spending in response to a high cholesterol diagnosis. They find that only those unaware of their high cholesterol and at high risk respond, but that the spending changes are only modest and temporary. Belot et al. (2020) test using an experiment whether providing either generic or tailored information on the risk of developing a heart attack or diabetes facilitates the adoption of healthier dietary habits. They find evidence of change in the short run, but predominantly for the generic treatment. They also test whether having additional time to choose has an effect on the healthiness of food choices, but no effect is found. Finally, Edwards (2018) looks at the impact of notification of results from submitted biomarkers on health behaviours. In particular, Edwards finds that notification of high blood glucose levels is associated with individual weight loss and increased exercise, as well as changes in the health behaviours of spouses.

A smaller related economics literature is on the impact of disease diagnoses, such as cancer, heart disease or diabetes, on dietary change (Fassier et al., 2017; Oster, 2018; Hut and Oster, 2022). One of the two papers in this area closest to mine, Oster (2018), investigates the impact of a diabetes diagnosis on diet changes and finds a small but significant effect on calorie reduction in the month right after diagnosis, though the effect is no longer significant in the months thereafter. The other closest related paper by Hut and Oster (2022) also find no effect of a diabetes diagnosis on diet, but rather that baseline diet and dietary concentration are the predictors of dietary change. My paper builds upon Oster (2018) and Hut and Oster (2022) by investigating the impact of diagnosis on several lifestyle behaviours, not only diet. By looking at several behaviours, and not just one, I allow for individuals to change certain behaviours but not others. This paper here differs from Oster (2018) by including heart attack, alongside diabetes, as the diagnosis being studied; Hut and Oster (2022) are more similar to my paper since they look at the diagnosis of three different disease categories, which include diabetes and heart disease. Finally, from a medical perspective Fassier et al. (2017) look at changes in diet and alcohol consumption before and after a cancer diagnosis. They find both healthy changes — decreased alcohol and sweetened drinks consumption — and unhealthy changes — decreased vegetable consumption and reduction in the intake of many vitamins and minerals.

This paper contributes to both the above literatures — diagnosis of risk factors and diagnosis of realized health risk — by looking at the receiving of a signal on health status via *both* the diagnosis of risk factors (high blood pressure and chest pain) and the diagnosis of the actual realized health risk (heart attack and diabetes). To the best of my knowledge, this is the first paper to compare the differences in response to a diagnosis of risk factors versus the diagnosis of a realized health risk.

Other related literature this paper ties into is on incorrect knowledge and uncertainty about the risks and risk factors for certain health conditions, such as heart attacks, and how they correspond to health status. Individuals may under or overestimate their perceived risk from engaging in certain lifestyle behaviours and hence also their subjective probabilities of having or getting a health condition or disease. For example, Belot et al. (2020), a paper closely related to this one finds, using an experimental approach, that individuals are often too pessimistic about their health status and thus the provision of tailored health information does not lead to improvements in lifestyle behaviours. For examples of incorrectly perceived risks for the different lifestyles, see: diet (Condon and McCarthy, 2006), smoking (Heikkinen et al., 2010), and exercise (Fitgerald et al., 1994; Crombie et al., 2004). For alcohol consumption, incorrectly perceived risks may stem from conflicting recommendations between some public health associations, such as the American Heart Association (2014) and the current medical literature (Stockwell et al., 2016), a likely source of confusion to the public and doctors alike. Similarly, Hurd and McGarry (2002) find that individuals respond to new information, such as the onset of a disease or other health condition (which can be considered a more precise signal), by updating their subjective probabilities of survival. The lack of clear signals on health status is one of the reasons why individuals may not be willing to make any changes to their behaviour (Sanderson et al., 2009; Logie-MacIver et al., 2012).

In the medical literature, for example, Condon and McCarthy (2006) find that some individuals believed heart attacks only occurred in older people and therefore thought they could postpone changing their lifestyle to a later time. They also find heart attack patients had already been aware of their poor lifestyles — whether it be smoking, stress or poor diet — and yet, for a variety of reasons and beliefs, many did not change their lifestyle prior to the event. Many individuals were waiting for an initial "warning sign" in order to motivate themselves to improve their lifestyle (Condon and McCarthy, 2006). Similar to the economics literature, this need for a warning sign can be interpreted as a need to receive a more precise signal on an individual's health status before knowing what is the optimal level of a lifestyle (change) to implement.

3 Power Calculations

Given the relatively small number of treated individuals in my sample, for many of my regression results there is a concern of insufficient power. This is particularly a problem when splitting the results further to get at heterogeneous effects such as sex or to assess the impact of the individual behaviours that make up the index.

The following section provides the minimum detectable effect (MDE) sizes that I expect given the data in order to assist in understanding whether insignificant results are due to insufficient power. If the effect size is smaller than the MDE then I cannot exclude that the effect is significant but only that the estimate is not precisely measured to due insufficient power. In contrast, if the estimate is larger than the MDE, then most likely the insignificant effect is actually a null result. For each regression presented in the paper the MDE size is shown in Table 17. I calculate the minimum detectable effect

size using the following equation:

$$MDE = (t_{\alpha/2} + t_{1-\kappa})\sqrt{\frac{\sigma^2}{np(1-p)}} = (t_{\alpha/2} + t_{1-\kappa})\frac{\sigma}{\sqrt{np(1-p)}}$$

Where using conventional values $t_{\alpha/2} = 1.96$ if $\alpha = 0.05$ and $t_{1-\kappa} = 0.84$ if $\kappa = 0.80$, n is the sample size, p is the proportion of the sample that is treated, and σ is the standard deviation (SD) of the dependent variable.

Table 17: Power Calculations

Table	Table Description	Dependent Variable	MDE
Table 4	Main realized health risk diagnosis	Index	0.304
Table 4	Main realized health risk diagnosis	Fruit/Veg	0.300
Table 4	Main realized health risk diagnosis	Walk 10	2.119
Table 4	Main realized health risk diagnosis	Walk 30	2.014
Table 4	Main realized health risk diagnosis	Smoke	0.048
Table 4	Main realized health risk diagnosis	Nr Cigs	3.036
Table 4	Main realized health risk diagnosis	Drink	0.056
Table 4	Main realized health risk diagnosis	Heavy	0.775
Table 4	Main realized health risk diagnosis	Days	0.336
Table 7	Realized health risk diagnosis only	Index	0.479
Table 7	Realized health risk diagnosis only	Fruit/Veg	0.467
Table 7	Realized health risk diagnosis only	Walk 10	3.330
Table 7	Realized health risk diagnosis only	Walk 30	3.196
Table 7	Realized health risk diagnosis only	Smoke	0.077
Table 7	Realized health risk diagnosis only	Nr Cigs	4.097
Table 7	Realized health risk diagnosis only	Drink	0.084
Table 7	Realized health risk diagnosis only	Heavy	1.253
Table 7	Realized health risk diagnosis only	Days	0.516
Table 8	Risk factor diagnosis only	Index	0.221
Table 8	Risk factor diagnosis only	Fruit/Veg	0.215
Table 8	Risk factor diagnosis only	Walk 10	1.534
Table 8	Risk factor diagnosis only	Walk 30	1.473
Table 8	Risk factor diagnosis only	Smoke	0.035
Table 8	Risk factor diagnosis only	Nr Cigs	2.040
Table 8	Risk factor diagnosis only	Drink	0.084
Table 8	Risk factor diagnosis only	Heavy	0.574
Table 8	Risk factor diagnosis only	Days	0.239
Table 5	Main realized h. risk diagnosis (only females)	Index	0.445
Table 5	Main realized h. risk diagnosis (only females)	Fruit/Veg	0.441
Table 5	Main realized h. risk diagnosis (only females)	Walk 10	3.092
Table 5	Main realized h. risk diagnosis (only females)	Walk 30	2.914
Table 5	Main realized h. risk diagnosis (only females)	Smoke	0.070
Table 5	Main realized h. risk diagnosis (only females)	Nr Cigs	3.962
Table 5	Main realized h. risk diagnosis (only females)	Drink	0.088

Continued on next page

Table 17 – *Continued from previous page*

Table	Table Description	Dependent Variable	MDE
Table 5	Main realized h. risk diagnosis (only females)	Heavy	1.034
Table 5	Main realized h. risk diagnosis (only females)	Days	0.476
Table 6	Main realized h. risk diagnosis (only males)	Index	0.419
Table 6	Main realized h. risk diagnosis (only males)	Fruit/Veg	0.410
Table 6	Main realized h. risk diagnosis (only males)	Walk 10	2.924
Table 6	Main realized h. risk diagnosis (only males)	Walk 30	2.812
Table 6	Main realized h. risk diagnosis (only males)	Smoke	0.066
Table 6	Main realized h. risk diagnosis (only males)	Nr Cigs	4.703
Table 6	Main realized h. risk diagnosis (only males)	Drink	0.067
Table 6	Main realized h. risk diagnosis (only males)	Heavy	1.179
Table 6	Main realized h. risk diagnosis (only males)	Days	0.481

4 Propensity Score Estimation Conditions

The propensity score is the probability an individual is treated given a set of selected observables. For proper application two lemmas must hold. First, the balancing property: observations with the same propensity score have the same distribution of observable covariates independently of treatment status. Second, the unconfoundedness assumption: the assignment to treatment is independent given the propensity score (Rosenbaum and Rubin, 1985). Finally, there must be common support between treated and control units.

To ensure that the balancing property is satisfied, after estimating the propensity score, the propensity score estimation program takes the full sample of treated and controls, sorts the individuals by their estimated propensity score, and divides them into bins such that within each bin the mean propensity score is not statistically different between the treated and controls groups. Similarly, the balancing property also requires that the mean of each covariate used in the estimation of the propensity score is balanced within each bin between treated and control groups. I choose the exact specification for the propensity score estimation such as to meet these requirements. Finally, I confirm common support by looking at the overlap in estimated propensity scores between treated and control units.

Main Analysis The balancing property is satisfied using seven bins. It is clear from Figure 1 that there is common support between treated and control units, as none of the treated units are marked as "off-support". Common support is further verified by Table 18, which shows the descriptive statistics of the estimated propensity score for the full sample, only the treated, and only the controls. It is important to note that both the minimum and the maximum for the treated group fall within the minimum and maximum of the control group. Therefore, common support for all treated units is further verified.

Realized Health Risk Diagnosis Only The estimated propensity score for the "realized diagnosis only" case satisfies the balancing property and has common support, shown in Figure 2, since once

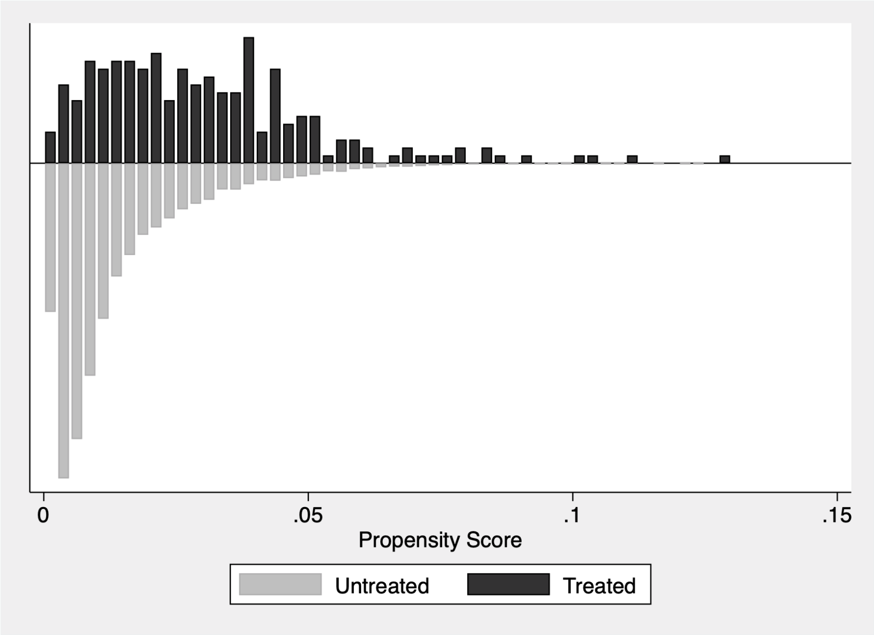

Figure 1: Estimated Propensity Score Histogram (Full Sample)

Table 18: Descriptive Statistics of Estimated Propensity Score (Full Sample)

Sample	Count	Mean	SD	Min	Max
Full Sample	22,158	0.0160	0.0157	0.00055	0.1446
Only Treated	354	0.0316	0.0226	0.00114	0.1286
Only Controls	21,804	0.0157	0.0154	0.00055	0.1446

again there are no treated units marked as off-support. Table 19, analogous to Table 18, shows the

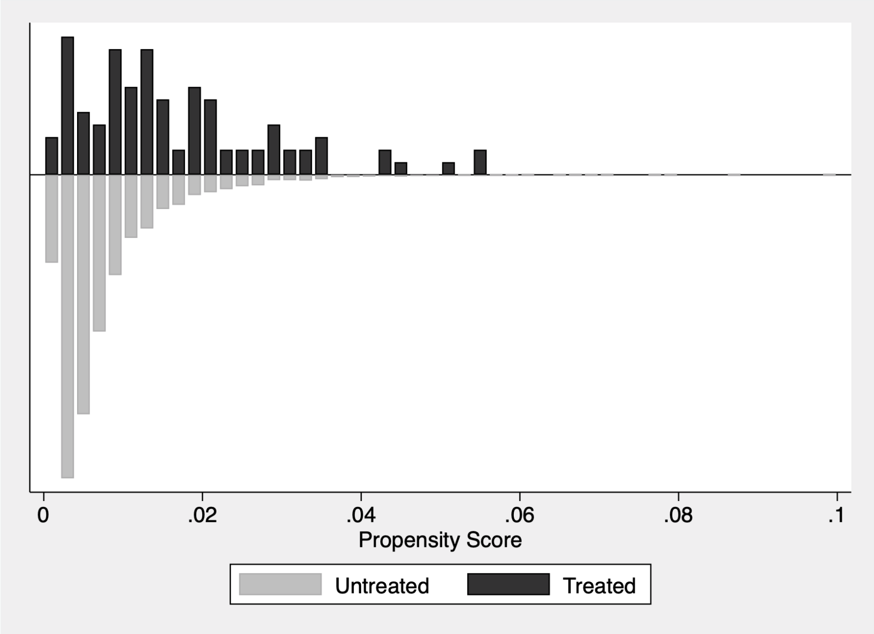

Figure 2: Estimated Propensity Score Histogram (Realized Health Risk Diag. Only)

descriptive statistics of the estimated propensity score for the three (sub)samples. Here as well, both

the minimum and the maximum for the treated group fall within the minimum and maximum of the control group, thereby reinforcing that all treated units fall within the common support.

Table 19: Descriptive Statistics of Estimated Propensity Score (Realized Health Risk Diagnosis Only)

Sample	Count	Mean	SD	Min	Max
Full Sample	17,155	0.0090	0.0089	0.00069	0.1084
Only Treated	154	0.0178	0.0143	0.00111	0.0795
Only Controls	17,001	0.0089	0.0088	0.00069	0.1084

Risk Factor Diagnosis Only The estimated propensity score for the "risk factor diagnosis only" variable satisfies the balancing property; and it has common support for all observations, as shown in Figure 3. Table 20 shows the descriptive statistics of the estimated propensity score for the full,

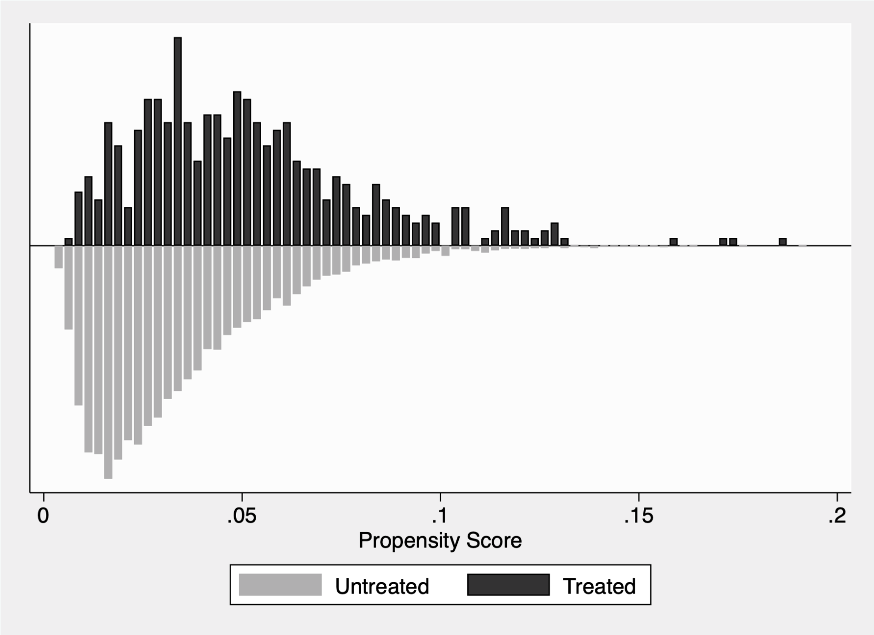

Figure 3: Estimated Propensity Score Histogram (Risk Factor Diagnosis Only)

treated-only, and control-only samples. Again, note that both the minimum and the maximum for the treated group fall within those of the control group, which confirms that the treated units fall within the common support.

Table 20: Descriptive Statistics of Estimated Propensity Score (Risk Factor Diagnosis Only)

Sample	Count	Mean	SD	Min	Max
Full Sample	17,625	0.0354	0.0249	0.00263	0.1967
Only Treated	624	0.0519	0.0297	0.00697	0.1869
Only Controls	17,001	0.0348	0.0244	0.00263	0.1967

5 Matching Strategy: Selection and Assessment

Due to the nature of matching, the propensity score is estimated and the matching performed *prior* to the analysis of the outcome variables. As a result, it is possible and encouraged to try several matching possibilities — such as nearest neighbour with or without replacement and with one or more controls, and radius or kernel with different bandwidths — and to run a range of balance diagnostics to assess which matching strategy leads to the best balance; this strategy is then used for the analysis of the outcomes (Garrido et al., 2014). The goal behind matching is to ensure that treated and control units are as similar as possible across observable covariates. Below are the selection and assessment criteria for both the main and secondary analyses.

5.1 Main Analysis

Selecting Matching Strategy with Best Balance Table 21 reports several possible matching strategies. In all cases, there are 232 treated individuals with a differing number of individuals used as controls depending on the matching strategy. Pairs are created for those individuals who are not missing the index outcome variable. There are only 232 treated observations and not the 354 reported in Table 18. This is because the "missing" individuals are missing one or more of the eight lifestyle index behaviour variables in either wave 2 or 5. As shown in Table 21 the kernel matching strategy with its selected bandwidth is the most balanced of the matches tried; the kernel matching with a bandwidth of 0.0075 is the best balanced match among the different kernel bandwidths tried. The kernel matching has the lowest mean and median percent standardized difference in covariates and the lowest Pseudo R^2. Furthermore, both the Rubin's B and Rubin's R fall within their desired cut-offs or ranges: below 25% and between [0.5,2], respectively. Therefore, I use this kernel matching strategy in the analysis. Below is a discussion of each summary measure of match quality and the characteristics of a good match. A comparison of the sensitivity of the findings to different matching strategies is provided in Matching and Weighting Strategies.

Table 21: Summary Measures of Match Quality for Original, Matched and Weighted Samples

Sample Type	Total Obs.	Total Treated	Total Controls	Pseudo R^2	Mean Standardized Difference (%)	Median Standardized Difference (%)	Rubin's B	Rubin's R
Original Sample	15,853	232	15,621	0.078	14.8	8.1	92.9*	0.92
NN 1:1 no replace.	464	232	232	0.033	5.7	4.7	42.9*	1.05
NN 1:1 with replace.	461	232	229	0.030	5.5	4.3	41.3*	1.04
Radius	15,853	232	15,621	0.013	4.3	3.2	26.5*	0.81
**Kernel**	**15,853**	**232**	**15,621**	**0.011**	**3.6**	**2.6**	**24.3**	**0.77**

Note: NN: Nearest Neighbour matching. Radius method has a caliper of 0.01. NN 1:1 with caliper omitted since match quality results are the same as the no caliper case. Kernel matching uses a bandwidth of 0.0075. If B>25% or R outside [0.5,2], marked with *.

Summary Measures of Match Quality Table 22 provides several summary measures of the overall balance of the variables used to estimate the propensity score and create the match, for both the unmatched and matched samples. The first column, the Pseudo-R^2, is the estimate from a probit of the

propensity score equation. The closer the value is to zero the more the variables used to estimate the propensity score no longer have predictive power for the realized health risk (or risk factor) diagnosis, which implies better balance. Similarly, the second column shows the p-value for the likelihood ratio test that all covariates used for the estimation are jointly insignificant. Both these columns suggest that the match between treated and controls is quite balanced. The third and fourth columns show that the mean and median percent standardized difference between the treatment and controls groups have been reduced for a large extent by matching, respectively; the mean percent decreases from 14.8 to 3.6, the median from 8.1 to 2.6. A mean percent standardized difference of less than 10% is considered a good quality match, which is what I find. Finally, the last two columns are summary measures of matching quality suggested by Rubin (2001). Rubin's B is the absolute standardized difference of the means of the propensity score of the treated and control group. Rubin's R is the variance ratio of the treated and control groups' propensity score. Rubin (2001) specifies that groups are sufficiently balanced when the Rubin's B is less than 25%; similarly, the Rubin's R should be between 0.5 and 2. Both Rubin measures are within the desired range, indicating a good match. Summarizing, overall Table 22 suggests the match is well balanced. A graphical interpretation of balance before and after matching at the individual covariate level is shown and discussed below.

Table 22: Summary Measures of Match Quality

Sample	Pseudo R^2	Likelihood ratio test p-value	Mean Standardized Difference (%)	Median Standardized Difference (%)	Rubin's B	Rubin's R
Unmatched	0.078	0.000	14.8	8.1	92.9*	0.92
Matched	0.011	1.000	3.6	2.6	24.3	0.77

Note: * if B>25%, R outside [0.5; 2]

Match Quality of Individual Covariates Figure 4 shows graphically the percent standardized difference (bias) for the covariates used in the propensity score estimation, both before and after matching. Overall, the figure shows that, except for ethnicity, the bias for all variables shown decreases with matching, often quite substantially (non-binary categorical variables are not shown for reasons of readability). In the case of ethnicity, the treated and untreated groups are not statistically different from each other in both the unmatched and matched cases, which means the increase is not of concern.

5.2 Secondary Analysis: Realized Health Risk Diagnosis Only

Selection Matching Strategy with Best Balance Among the reported matching strategies, in Table 23, the kernel matching strategy has the lowest mean and median percent standardized difference in covariates and the lowest Pseudo R^2. For the Rubin's B, although it does not fall within the desired 25% cut-off, the kernel strategy has the lowest Rubin's B value of all the matching strategies. Finally, the Rubin's R falls within the desired range of [0.5,2]. Although the kernel match is still not very well-balanced — as suggested by both the Rubin's B and the higher values of the other measures compared to both the main analysis match (shown previously) and the risk factor diagnosis only analysis

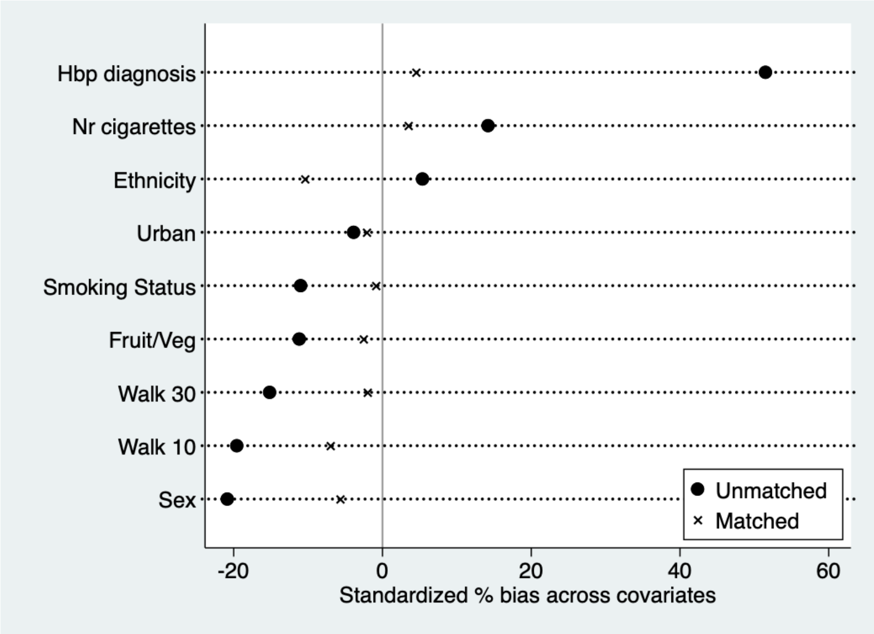

Figure 4: Percent Standardized Difference (Bias) Across Propensity Score Estimation Covariates Before and After Matching

match (shown subsequently) — the kernel strategy is still the best choice of the available strategies and therefore I use it for this realized health risk diagnosis only analysis.

Table 23: Summary Measures of Match Quality for Original, Matched and Weighted Samples (Realized Health Risk Diagnosis Only)

Sample Type	Total Obs.	Total Treated	Total Controls	Pseudo R^2	Mean Standardized Difference (%)	Median Standardized Difference (%)	Rubin's B	Rubin's R
Original Sample	12,339	94	12,245	0.063	13.7	8.5	84.9*	1.27
NN 1:1 no replace.	188	94	94	0.058	8.0	6.0	57.4*	1.14
NN 1:1 with replace.	187	94	93	0.059	8.0	6.1	57.9*	1.11
Radius	12,329	94	12,235	0.035	8.4	7.6	44.6*	0.76
**Kernel**	**12,323**	**94**	**12,229**	**0.025**	**5.7**	**4.8**	**37.1***	**0.65**

Note: NN: Nearest Neighbour matching. Radius method has a caliper of 0.01. NN 1:1 with caliper omitted since match quality results are the same as the no caliper case. Kernel matching uses a bandwidth of 0.00375. If B>25% or R outside [0.5,2], marked with *.

Summary Matching Quality Assessment Table 24 provides, for the realized health risk diagnosis only case, several summary measures of the overall balance of the variables used to estimate the propensity score and create the match, for both the unmatched and matched samples. The measures are the same as those described previously for the main analysis case. In this realized health risk diagnosis only case, the measures indicate that matching improves the balance between treated and controls. For example, the average percent standardized differences of the mean and median for the covariates from the propensity score estimation decreases: the mean from 13.7 to 5.7 and the median from 8.5 to 4.8. However, the Rubin's B measure suggests that although the matched sample is an improvement over the unmatched one, it is still not very well-balanced as the value (37.1%) falls above the 25% threshold.

This less good match is likely in part attributable to the relatively small sample size of the treated for this realized health risk diagnosis only case compared to the other two cases. Nevertheless, the measures still suggest that the matched sample has more balance than the unmatched sample and therefore I use it for this “realized diagnosis only” analysis. A graphical interpretation of balance before and after matching at the individual covariate level is below.

Table 24: Summary Measures of Match Quality (Realized Health Risk Diagnosis Only)

Sample	Pseudo R^2	Likelihood ratio test p-value	Mean Standardized Difference (%)	Median Standardized Difference (%)	Rubin’s B	Rubin’s R
Unmatched	0.063	0.000	13.7	8.5	84.9*	1.27
Matched	0.025	1.000	5.7	4.8	37.1*	0.65

Note: * if B>25%, R outside [0.5;2]

Match Quality of Individual Covariates Figure 5 shows graphically the percent standardized difference (bias) for the covariates used in the propensity score estimation for the realized health risk diagnosis only, both before and after matching. In this case, the figure shows the matching is less successful in reducing the difference between the unmatched treated and unmatched control groups (i.e. the bias). For age and the interaction between sex and employment status matching reduces the bias quite drastically. However, for most other variables shown matching does not affect the bias much. Once again, non-binary categorical variables are not shown for readability reasons. However, none of the variables are statistically different between the treated and untreated groups after matching.

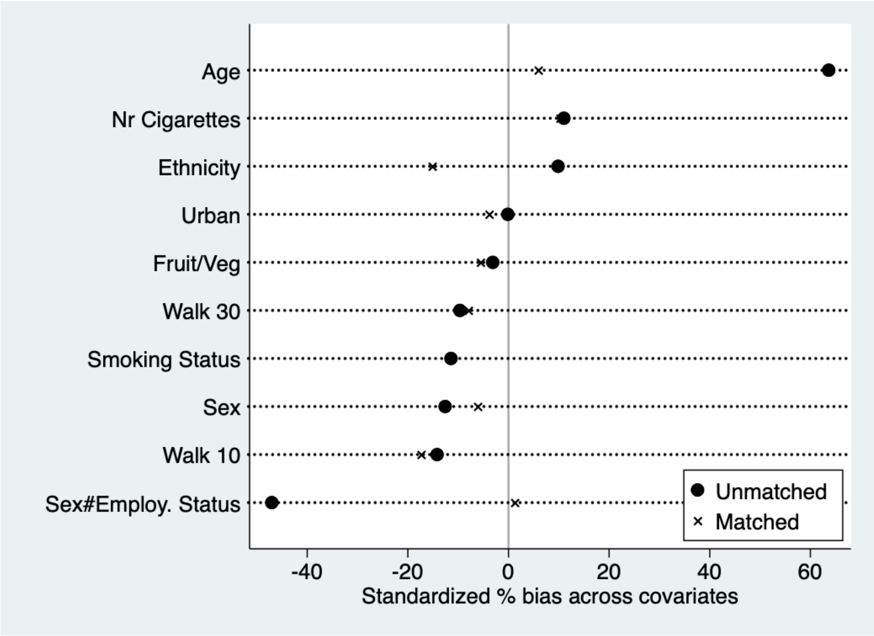

Figure 5: Percent Standardized Difference (Bias) Across Propensity Score Estimation Covariates Before and After Matching (Realized Health Risk Diagnosis Only)

5.3 Secondary Analysis: Risk Factor Diagnosis Only

Selection Matching Strategy with Best Balance Among the reported matching strategies in Table 25 kernel matching has the lowest mean and median percent standardized difference in covariates and the lowest Pseudo R^2. Furthermore, both the Rubin's B and Rubin's R fall within their desired cut-offs or ranges, below 25% and between [0.5,2], respectively. Therefore, I use this kernel matching strategy in the analysis.

Table 25: Summary Measures of Match Quality for Original, Matched and Weighted Samples (Risk Factor Diagnosis)

Sample Type	Total Obs.	Total Treated	Total Controls	Pseudo R^2	Mean Standardized Difference (%)	Median Standardized Difference (%)	Rubin's B	Rubin's R
Original Sample	12,701	456	12,245	0.052	12.0	6.3	71.2*	0.77
NN 1:1 no replace.	912	456	456	0.023	5.5	4.1	35.8*	1.50
NN 1:1 with replace.	895	456	439	0.022	5.3	4.5	34.9*	1.48
Radius	12,693	456	12,237	0.007	2.8	2.4	19.4	1.03
**Kernel**	**12,685**	**456**	**12,229**	**0.005**	**2.7**	**2.2**	**17.0**	**1.05**

Note: NN: Nearest Neighbour matching. Radius method has a caliper of 0.01. NN 1:1 with caliper omitted since match quality results are the same as the no caliper case. Kernel matching uses a bandwidth of 0.0075. If B>25% or R outside [0.5,2], marked with *.

Summary Match Quality Assessment Table 26 provides for the risk factor diagnosis case several summary measures of the overall balance of the variables used to estimate the propensity score and create the match, for both the unmatched and matched samples. The measures are the same as those described previously and all suggest a good match. Summarizing, overall Table 26 suggests a well-balanced match. A graphical interpretation of balance before and after matching at the individual covariate level is below.

Table 26: Summary Measures of Match Quality (Risk Factor Diagnosis Only)

Sample	Pseudo R^2	Likelihood ratio test p-value	Mean Standardized Difference (%)	Median Standardized Difference (%)	Rubin's B	Rubin's R
Unmatched	0.052	0.000	12.0	6.3	71.2*	0.77
Matched	0.005	1.000	2.7	2.2	17.0	1.05

Note: * if B>25%, R outside [0.5;2]

Match Quality of Individual Covariates Figure 6 shows graphically the percent standardized difference (bias) for the covariates used in the risk factor diagnosis only propensity score estimation, both before and after matching. Overall, the figure shows that except for Fruit/Veg the bias for all variables shown decreases with matching, often quite substantially. Once again, non-binary categorical variables are not shown for readability reasons. In the case of Fruit/Veg, the treated and untreated groups are not statistically different from each other in either the unmatched and matched cases; this slight increase is therefore of no concern.

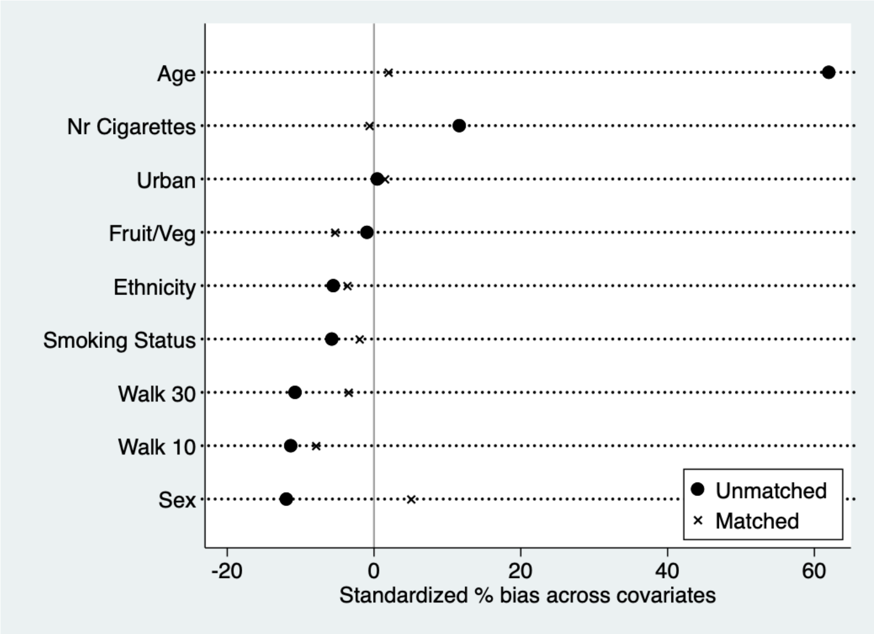

Figure 6: Percent Standardized Difference (Bias) Across Propensity Score Estimation Covariates Before and After Matching (Risk Factor Diagnosis Only)

6 Matching and Weighting Strategies

Stratification Stratification takes the full sample of treated and controls, sorts the individuals by estimated propensity score and then splits them into bins such that within each bin the mean propensity score is the same for the treated and controls groups. The analysis (the effect calculation) is performed for each bin with the assumption that within each bin individuals are similar.

Nearest Neighbour (NN) 1:1 Matching without Replacement Nearest neighbour 1:1 matching can be implemented with or without replacement (a caliper to restrict the distance from which the nearest control is selected has no effect). In the case of no replacement, for each treated individual the control with the closest propensity score is selected, except where that control has already been assigned to another treated individual, in that case the next nearest control unit is selected.

NN 1:1 Matching with Replacement Matching 1:1 with replacement is similar to matching 1:1 without replacement except that the control is returned to the pool each time, such that it can be used as a control for another treated individual. This means that for each treated individual the control with the closest propensity score is always selected.

NN 1:N Matching, N>1 Matching more than one control to the treated unit (1:N) is only possible with replacement. It is similar to matching 1:1 with replacement except that instead of matching a treated individual with its single nearest control, the treated individual is matched to its N nearest controls.

Kernel Matching Kernel matching uses all observations within the common support, taking a weighted average of the inverse of the (propensity score) distance between each treated and control unit. In other words, control units nearer to the treated unit receive more weight.

Radius Matching In radius matching for each treated unit within a specified radius all controls are used and assigned equal weight regardless of their (propensity score) distance to the treated unit.

6.1 Comparing Matching Strategies and Standard Errors

Table 27 compares several matching strategies as well as the different options for standard errors. The main purpose of this table is to show that neither the matching strategy nor the choice for standard errors have a significant impact on the final findings. None of the estimates are statistically significantly different from one another at the 5% level. In each column the estimate reported is the impact of the main analysis realized health risk diagnosis on the index, first differenced.

Column 1 presents the regression analysis matching using stratification and using regular robust standard errors. Column 2 presents the same as column 1 except that it clusters standard errors at the household level. From these two columns it is clear that such household-level clustering of standard errors has no effect on changing the reported standard errors.

When considering the matching strategy directly, rather than including matching as part of a regression analysis, there are several aspects to decide upon. First, the exact command to execute the matching (*psmatch*, *nnmatch* or *psmatch2*). Second, whether to provide the matching program/command with the propensity score directly (pscore) or whether to provide it with the covariates used to calculate the propensity score (cov) and allow the program to calculate its own propensity score that it will subsequently use to match on. One of the main advantages of propensity score estimation using the *pscore* matching program, over other programs, is as part of the estimation this program checks and requires that the estimated propensity score, as well as the covariates used in such an estimation, are balanced among propensity score bins of treated and control groups; something that I would otherwise have to check manually. Third, decide on what kind of standard errors to use, both in terms of what the default standard errors are for each matching program/command but also how the standard errors can be adjusted to account for the number of matches used (if using nearest neighbour, N>1) and/or to use bootstrap standard errors, where applicable. For *psmatch* and *nnmatch* the default is robust Abadie-Imbens (A-I) standard errors, which takes into account that the propensity score is estimated rather than known.

In columns 3-5 I match 1:1 using A-I standard errors. Column 3 uses the *psmatch* command and provides the matching program with the previously calculated propensity score directly. Column 4 uses the *psmatch* command but now I provide the program with the covariates to calculate its own propensity score prior to matching. Column 5 uses the same approach as column 4, but now using the *nnmatch* command. The *nnmatch* command only allows the provision of the covariates to calculate its own propensity score prior to matching; it does not allow the input of a previously calculated propensity score.

Finally, columns 6 and 7 use the *psmatch2* command, which allows the use of the kernel matching

strategy. Column 6 shows the effects using the default standard errors while column 7 shows the same effects but using bootstrap standard errors.

Summarizing, from this table neither the exact choice of matching strategy nor the standard error adjustment has any significant effect on the estimates or standard errors displayed. In general, the effect has a point estimate of approximately 0.23. The chosen specification is *psmatch2* with previously calculated propensity score, bandwidth of 0.0075 and bootstrap standard errors, corresponding to column 7. I already discussed the choice of optimal matching strategy in Matching Strategy: Selection and Assessment.

Table 11: Realized Health Risk Diagnosis on Index — Several Matching Strategies and Standard Errors

	(1)	(2)	(3)	(4)	(5)	(6)	(7)
	OLS stratification no hh cl se	OLS stratification hh cl se	psmatch (pscore) NN 1:1 A-I se	psmatch (cov) NN 1:1 A-I se	nnmatch (cov) NN 1:1 A-I se	psmatch2 (pscore) kernel 0.0075 bandwidth se	psmatch2 (pscore) kernel 0.0075 bandwidth bootstrap se (1000reps)
Realized Diag.	0.230**	0.230**					
	(0.111)	(0.111)					
ATT			0.231**	0.221	0.172	0.227***	0.227**
			(0.111)	(0.142)	(0.141)	(0.111)	(0.111)
Constant	0.028	0.028					
	(0.027)	(0.028)					
Observations	15,853	15,853	15,853	15,853	15,853	15,853	15,853

Standard errors in parentheses. See column headers for type of standard error (se). * $p < 0.1$, ** $p < 0.05$, *** $p < 0.01$

References

American Heart Association (2014). "Alcohol and Heart Health." URL https://www.heart.org/en/healthy-living/healthy-eating/eat-smart/nutrition-basics/alcohol-and-heart-health. Article date: 14 Aug, 2014.

Atkin, David (2016). "The caloric costs of culture: Evidence from Indian migrants." *American Economic Review*, 106, 1144–1181.

Bartiaux, Françoise (2008). "Does environmental information overcome practice compartmentalisation and change consumers' behaviours?" *Journal of Cleaner Production*, 16, 1170–1180.

Belot, Michèle, Jonathan James, and Jonathan Spiteri (2020). "Facilitating healthy dietary habits: An experiment with a low income population." *European Economic Review*, 129, 103550.

Benhabib, Jess, Alberto Bisin, and Andrew Schotter (2010). "Present-bias, quasi-hyperbolic discounting, and fixed costs." *Games and Economic Behavior*, 69(2), 205–223.

Bhalotra, Sonia, Adeline Delavande, Paul Fisher, and Jonathan James (2020). "The Impact of a Personalised Blood Pressure Warning on Health Outcomes and Behaviours." *Institute of Social and Economic Research (ISER) Working Paper Series; No. 2020-2.*

Brownell, Kelly D. and Lisa R. Cohen (1995). "Adherence to dietary regimens 2: Components of effective interventions." *Behavioral Medicine*, 20, 155–164.

Carrera, Mariana, Syeda A. Hasan, and Silvia Prina (2020). "Do health risk assessments change eating habits at the workplace?" *Journal of Economic Behaviour and Organization*, 172, 236–246.

Condon, Carol and Geraldine McCarthy (2006). "Lifestyle changes following acute myocardial infarction: Patients perspectives." *European Journal of Cardiovascular Nursing*, 5, 37–44.

Craig, Cora L., Catrine Tudor-Locke, and Adrian Bauman (2006). "Twelve-month effects of Canada on the

Move: A population-wide campaign to promote pedometer use and walking." *Health Education Research*, 22, 406–413.

Crombie, Iain K., Linda Irvine, Brian Williams, Alison R McGinnis, Peter W. Slane, Elizabeth M. Alder, and Marion E. T. McMurdo (2004). "Why older people do not participate in leisure time physical activity: A survey of activity levels, beliefs and deterrents." *Age and Ageing*, 33, 287–292.

Durkin, Sarah, Emily Brennan, and Melanie Wakefield (2012). "Mass media campaigns to promote smoking cessation among adults: An integrative review." *Tobacco Control*, 21, 127–138.

Edwards, Ryan D. (2018). "If My Blood Pressure Is High, Do I Take It to Heart? Behavioral Effects of Biomarker Collection in the Health and Retirement Study." *Demography*, 55, 403–434.

Esselstyn, Caldwell B., Jr., Stephen G. Ellis, Sharon V. Medendorp, and Timothy D. Crowe (1995). "A strategy to arrest and reverse coronary artery disease: A 5-year longitudinal study of a single physician's practice." *Journal of Family Practice*, 41, 560–568.

Fadlon, Itzik and Torben Heien Nielsen (2019). "Family health behaviors." *American Economic Review*, 109, 3162–3191.

Fassier, Philippine, Laurent Zelek, Lucie Lécuyer, Patrick Bachmann, Marina Touillaud, Nathalie Druesne-Pecollo, Pilar Galan, Patrice Cohen, Hélène Hoarau, Paule Latino-Martel, Emmanuelle Kesse-Guyot, Julia Baudry, Serge Hercberg, Mélanie Deschasaux, and Mathilde Touvier (2017). "Modifications in dietary and alcohol intakes between before and after cancer diagnosis: Results from the prospective population-based NutriNet-Santé cohort." *International Journal of Cancer*, 141, 457–470.

Fitgerald, James T., Steven P. Singleton, Anne Victoria Neale, Ananda S. Prasad, and Joseph W. Hess (1994). "Activity levels, fitness status, exercise knowledge, and exercise beliefs among healthy, older African American and white women." *Journal of Aging and Health*, 6, 296–313.

Flay, Brian R. (1987). "Mass media and smoking cessation: A critical review." *American Journal of Public Health*, 77, 153–160.

Fleming, Michael F., Linda Baier Manwell, Kristen Lawton Barry, Wendy Adams, and Ellyn A. Stauffacher (1999). "Brief physician advice for alcohol problems in older adults: A randomized community-based trial." *Journal of Family Practice*, 48, 378–386.

Garrido, Melissa M., Amy S. Kelley, Julia Paris, Katherine Roza, Diane E. Meier, R. Sean Morrison, and Melissa D. Aldridge (2014). "Methods for constructing and assessing propensity scores." *Health Services Research*, 49, 1701–1720.

Heikkinen, Hanne, Kristiina Patja, and Piia Jallinoja (2010). "Smokers' accounts on the health risks of smoking: Why is smoking not dangerous for me?" *Social Science & Medicine*, 71, 877–883.

Hurd, Michael D and Kathleen McGarry (2002). "The predictive validity of subjective probabilities of survival." *The Economic Journal*, 112(482), 966–985.

Hut, Stefan and Emily Oster (2022). "Changes in household diet: Determinants and predictability." *Journal of Public Economics*, 208.

Kelly, Michael P and Mary Barker (2016). "Why is changing health-related behaviour so difficult?" *Public Health*, 136, 109–116.

Laibson, David (1997). "Golden eggs and hyperbolic discounting." *The Quarterly Journal of Economics*, 112(2), 443–478.

Lanza, Elaine, Arthur Schatzkin, Cassandra Daston, Don Corle, Laurence Freedman, Rachel Ballard-Barbash, Bette Caan, Peter Lance, James Marshall, Frank Iber, Moshe Shike, Joel Weissfeld, Martha Slattery, Electra Paskett, Donna Mateski, Paul Albert, and the PPT Study Group (2001). "Implementation of a 4-y, high-fiber,

high-fruit-and-vegetable, low-fat dietary intervention: Results of dietary changes in the Polyp Prevention Trial." *American Journal of Clinical Nutrition*, 74, 387–401.

Larimer, Mary E. and Jessica M. Cronce (2002). "Identification, prevention and treatment: A review of individual-focused strategies to reduce problematic alcohol consumption by college students." *Journal of Studies on Alcohol. Supplement*, s14, 148–163.

Logie-MacIver, Liz, Maria Piacentini, and Douglas Eadie (2012). "Using qualitative methodologies to understand behaviour change." *Qualitative Market Research: An International Journal*, 15, 70–86.

Mathis, Klaus and Ariel David Steffen (2015). "From rational choice to behavioural economics." In *European Perspectives on Behavioural Law and Economics*, edited by Klaus Mathis, pp. 31–48. Springer.

O'Donoghue, Ted and Matthew Rabin (1999). "Doing it now or later." *American Economic Review*, 89(1), 103–124.

O'Donoghue, Ted and Matthew Rabin (2015). "Present bias: Lessons learned and to be learned." *American Economic Review*, 105(5), 273–79.

Ornish, Dean, Larry W. Scherwitz, James H. Billings, K. Lance Gould, Terri A. Merritt, Stephen Sparler, William T. Armstrong, Thomas A. Ports, Richard L. Kirkeeide, Charissa Hogeboom, and Richard J. Brand (1998). "Intensive Lifestyle Changes for Reversal of Coronary Heart Disease." *Journal of the American Medical Association*, 280, 2001–2007.

Oster, Emily (2018). "Diabetes and diet: Purchasing behavior change in response to health information." *American Economic Journal: Applied Economics*, 10, 308–348.

Rosenbaum, Paul R. and Donald B. Rubin (1985). "Constructing a control group using multivariate matched sampling methods that incorporate the propensity score." *The American Statistician*, 39, 33–38.

Rubin, Donald B. (2001). "Using propensity scores to help design observational studies: Application to the tobacco litigation." *Health Services and Outcomes Research Methodology*, 2(3-4), 169–188.

Sanderson, Saskia C., Jo Waller, Martin J. Jarvis, Steve E. Humphries, and Jane Wardle (2009). "Awareness of lifestyle risk factors for cancer and heart disease among adults in the UK." *Patient and Counseling*, 74, 221–227.

Stockwell, Tim, Jinhui Zhao, Sapna Panwar, Audra Roemer, Timothy Naimi, and Tanya Chikritzhs (2016). "Do "moderate" drinkers have reduced mortality risk? A systematic review and meta-analysis of alcohol consumption and all-cause mortality." *Journal of Studies on Alcohol and Drugs*, 77, 185–198.

Strecher, Victor J., Matthew Kreuter, Dirk-Jan Den Boer, Sarah Kobrin, Harm J. Hospers, and Celette S. Skinner (1994). "The effects of computer-tailored smoking cessation messages in family practice settings." *Journal of Family Practice*, 39, 262–270.

Worsley, Anthony (2002). "Nutrition knowledge and food consumption: Can nutrition knowledge change food behaviour." *Asia Pacific Journal of Clinical Nutrition*, 11(S6), S579–S585.

Young, Deborah Rohm, William L Haskell, C Barr Taylor, and Stephen P Fortmann (1996). "Effect of community health education on physical activity knowledge, attitudes, and behavior: The Stanford Five-City Project." *American Journal of Epidemiology*, 144, 264–274.

Zhao, Meng, Yoshifumi Konishi, and Paul Glewwe (2013). "Does information on health status lead to a healthier lifestyle? Evidence from China on the effect of hypertension diagnosis on food consumption." *Journal of Health Economics*, 32, 367–385.
